# Immuno-PET Imaging of CD69 Visualizes T-Cell Activation and Predicts Survival Following Immunotherapy in Murine Glioblastoma

**DOI:** 10.1158/2767-9764.CRC-22-0434

**Published:** 2023-07-06

**Authors:** Michal Nisnboym, Sarah R. Vincze, Zujian Xiong, Chaim T. Sneiderman, Rebecca A. Raphael, Bo Li, Ambika P. Jaswal, ReidAnn E. Sever, Kathryn E. Day, Joseph D. LaToche, Lesley M. Foley, Hanieh Karimi, T. Kevin Hitchens, Sameer Agnihotri, Baoli Hu, Dhivyaa Rajasundaram, Carolyn J. Anderson, Deborah T. Blumenthal, Thomas M. Pearce, Shikhar Uttam, Jessie R. Nedrow, Ashok Panigrahy, Ian F. Pollack, Frank S. Lieberman, Jan Drappatz, Itay Raphael, Wilson B. Edwards, Gary Kohanbash

**Affiliations:** 1 Department of Neurological Surgery, UPMC Children's Hospital of Pittsburgh, University of Pittsburgh School of Medicine, Pittsburgh, Pennsylvania.; 2 Department of Neurology, Tel-Aviv Sourasky Medical Center, Tel-Aviv University, Tel-Aviv, Israel.; 3 Department of Computational and Systems Biology, UPMC Hillman Cancer Center, Cancer Biology Program, University of Pittsburgh, Pittsburgh, Pennsylvania.; 4 In Vivo Imaging Facility, University of Pittsburgh Medical Center, UPMC Hillman Cancer Center, Pittsburgh, Pennsylvania.; 5 Department of Biochemistry, University of Missouri, Columbia, Missouri.; 6 Department of Neurobiology, University of Pittsburgh School of Medicine, Pittsburgh, Pennsylvania.; 7 Division of Health Informatics, Department of Pediatrics, UPMC Children's Hospital of Pittsburgh, University of Pittsburgh School of Medicine, Pittsburgh, Pennsylvania.; 8 Department of Chemistry, University of Missouri, Columbia, Missouri.; 9 Neuro-oncology Division, Tel-Aviv Sourasky Medical Center, Tel-Aviv University, Tel-Aviv, Israel.; 10 Division of Neuropathology, Department of Pathology, University of Pittsburgh School of Medicine, Pittsburgh, Pennsylvania.; 11 Department of Radiology, UPMC Children's Hospital of Pittsburgh, University of Pittsburgh Medical Center, Pittsburgh, Pennsylvania.; 12 Neuro-oncology Program, Division of Hematology/Oncology, UPMC Hillman Cancer Center, Pittsburgh, Pennsylvania.; 13 Department of Immunology, University of Pittsburgh, Pittsburgh, Pennsylvania.

## Abstract

Glioblastoma (GBM) is the most common and malignant primary brain tumor in adults. Immunotherapy may be promising for the treatment of some patients with GBM; however, there is a need for noninvasive neuroimaging techniques to predict immunotherapeutic responses. The effectiveness of most immunotherapeutic strategies requires T-cell activation. Therefore, we aimed to evaluate an early marker of T-cell activation, CD69, for its use as an imaging biomarker of response to immunotherapy for GBM. Herein, we performed CD69 immunostaining on human and mouse T cells following *in vitro* activation and post immune checkpoint inhibitors (ICI) in an orthotopic syngeneic mouse glioma model. CD69 expression on tumor-infiltrating leukocytes was assessed using single-cell RNA sequencing (scRNA-seq) data from patients with recurrent GBM receiving ICI. Radiolabeled CD69 Ab PET/CT imaging (CD69 immuno-PET) was performed on GBM-bearing mice longitudinally to quantify CD69 and its association with survival following immunotherapy. We show CD69 expression is upregulated upon T-cell activation and on tumor-infiltrating lymphocytes (TIL) in response to immunotherapy. Similarly, scRNA-seq data demonstrated elevated CD69 on TILs from patients with ICI-treated recurrent GBM as compared with TILs from control cohorts. CD69 immuno-PET studies showed a significantly higher tracer uptake in the tumors of ICI-treated mice compared with controls. Importantly, we observed a positive correlation between survival and CD69 immuno-PET signals in immunotherapy-treated animals and established a trajectory of T-cell activation by virtue of CD69-immuno-PET measurements. Our study supports the potential use of CD69 immuno-PET as an immunotherapy response assessment imaging tool for patients with GBM.

**Significance:**

Immunotherapy may hold promise for the treatment of some patients with GBM. There is a need to assess therapy responsiveness to allow the continuation of effective treatment in responders and to avoid ineffective treatment with potential adverse effects in the nonresponders. We demonstrate that noninvasive PET/CT imaging of CD69 may allow early detection of immunotherapy responsiveness in patients with GBM.

## Introduction

Glioblastoma (GBM) is the most malignant brain tumor in adults, with a median overall survival (OS) of 14–16 months from initial diagnosis despite aggressive multimodal therapy (1, 2). The current standard of care for GBM comprises maximal safe surgical resection followed by concurrent chemo radiotherapy (CCRT), maintenance chemotherapy with temozolomide (3), and tumor-treating fields (TTF; ref. 4). Because of the aggressive and infiltrative nature of GBMs, tumor recurrence is inevitable after initial therapy (5). At recurrence, treatment options are largely palliative and associated with only a partial response and variable survival benefits (6).

To identify an effective treatment for brain tumors, several immunotherapeutic approaches have been introduced to harness the patient's immune response to fight and eliminate tumor cells. Although immune checkpoint inhibitor (ICI)-based immunotherapy has revolutionized the treatment of several cancers including metastatic melanoma (7), non–small cell lung cancer (8), and renal cell carcinoma (9), the objective response rates for these cancers are only up to approximately 50% (10). ICIs are mAbs that block proteins present on T cells from binding to inhibitory ligands present on tumor cell surfaces, thereby preventing tumor cells from suppressing the activity of cytotoxic T cells and resulting in a profound inflammatory response within the tumor bed. The key players in inhibitory checkpoint signaling pathways include programmed cell death protein-1 (PD-1) and cytotoxic T-lymphocyte-associated protein-4 (CTLA-4; ref. 11).

In many preclinical studies on glioma-bearing mice, ICI improved survival outcomes and elicited antitumor immunity (12–16). As such, ICI have been used in several large multicenter early- and late-phase clinical trials and have yielded mixed results (13–17). Many clinical studies conducted to date have found subgroups of patients, albeit small, with a long-lasting clinical benefit from ICI treatment (10, 18). Notably, a recent randomized, multi-institutional clinical trial reported that patients treated with neoadjuvant pembrolizumab (anti–PD-1) had significantly prolonged OS and progression-free survival (PFS; ref. 19).

Although progress has been made in the development of immunotherapies, the available imaging tools capable of quantifying and monitoring responses to these therapies remain limited in the clinical field. Clinical MRI is the mainstay for assessing treatment response to therapy in neuro-oncology. However, it exhibits morphologic features and is thus nonspecific (20). For most neuro-oncology treatments, including ICI, patients continue treatment until radiographic evidence of tumor progression by MRI (21, 22). Consequently, nonresponding patients who would otherwise have the opportunity to attempt potentially more effective treatment regimens may spend most of their remaining months on ineffective treatment and might experience severe side effects. Hence, there is a need to develop novel and more effective imaging strategies that provide molecular information and determine early on whether a patient responds to immunotherapy.

PET is a unique nuclear medicine modality that enables the real-time imaging quantification of molecular processes by administering a radiolabeled tracer that attaches a high-affinity peptide or protein to a cell surface receptor. The most commonly used tracer is fluorine-18-fluorodeoxyglucose (^18^F -FDG). FDG-PET is an imaging method based on the increased rate of glucose metabolism (23, 24) and is sufficient for monitoring therapeutic effects in most malignancies; however, its role in brain oncology is limited, as it exhibits low specificity due to its accumulation in all hyper metabolic cells, specifically glucose-avid normal brain cells (25).

Recently, several studies have focused on engineering new tracers, using biologics labeled with long half-life radioisotopes such as Zr-89 (78.4 hours; ref. 26) and targeting checkpoint molecules such as PD-1/programmed death-ligand 1 (PD-L1)/cytotoxic T-lymphocyte associated protein 4 (CTLA-4) to predict the response to ICI in murine models (27) and clinical trials (28, 29). However, while in a clinical trial evaluating PD-L1 immuno-PET, standardized uptake value (SUV) showed a correlation with response, the signal uptake in tumors was heterogeneous, varying within and among lesions, patients, and tumor types (29). Moreover, emerging varieties of cancer immunotherapies highlight the need for the direct evaluation of immune cells.

PET imaging probes that selectively depict effector T cells, which act as key players in tumor eradication, may be valuable for therapeutic assessment. PET antibodies that target effector immune cells are currently being evaluated in clinical trials. In a previous study (30), patients with metastatic solid tumors undergoing ICI therapy underwent PET imaging with an anti-CD8 radiolabeled minibody and CD8^+^ tumor-infiltrating lymphocytes (TIL) were detected in tumors 24 hours postinfusion. However, one known limitation of imaging CD8 to examine antitumor immunity is that many CD8^+^ TILs can be bystander cells, which likely have little effect on the clinical response (31).

Furthermore, a group from Stanford University reported successful developments in immune system visualization techniques using OX40 mAb for imaging OX40^+^ activated T cells in a mouse model of syngeneic lymphoma (32) and ^89^Zr-DFO-ICOS mAb tracer, for detecting ICOS^+^ T cells in a murine lung cancer model (33). Following these promising results, they extended their pursuit to the more challenging type of tumor, GBM, and demonstrated that ^89^Zr-DFO-OX40 mAb PET specifically delineates stimulated lymphoid organs following the administration of a cancer vaccine applied in an orthotopic GBM model. However, direct evaluation of intratumoral lymphocytes between responders and nonresponders was unachievable; therefore, they could not corroborate the response resulting from the migrated cytotoxic T cells (34).

Cluster of differentiation 69 (CD69), a C-type lectin protein, is one of the earliest cell surface proteins expressed by activated lymphocytes (35–37). It is also involved in lymphocyte proliferation and functions as a signal-transmitting receptor (38). CD69 is expressed by mature activated T cells and platelets and is not found in resting circulating leukocytes in humans (37). It is also a marker for tissue-resident memory T-cells (predominantly outside the CNS; ref. 39). Although several other immune cell populations, including natural killer (NK) cells, can express CD69 (40–45), it is an explicit T-cell activation hallmark. Thus, CD69 is a general surface marker for activated immune cells in tissues with limited presence in the circulation, and its specific functional and fundamental expression on T cells suggests that it could act as a biomarker of activated antitumor T cells. This is further supported by recent work on a murine colon carcinoma model that showed radiolabeled CD69-directed Ab combined with PET imaging as a noninvasive method to assess the early response to ICI with increased uptake in the tumors and lymphoid tissue of ICI-responsive tumor-bearing mice (46).

In this study, we utilized Ab-based PET/CT imaging (immuno-PET) with ^89^Zr-DFO-CD69 Ab to achieve noninvasive observations of the immune response in preclinical GBM. We demonstrate the feasibility of early visualization and quantification of T-cell activation in response to ICI immunotherapy in a murine GBM model, with a positive correlation with survival. Together with scRNA-seq data of recurrent GBM tissues from patients receiving versus not receiving ICI, our study shows the potential incorporation of CD69 immuno-PET as a response assessment to immunotherapy in patients with GBM.

## Materials and Methods

### T-Cell Activation

Jurkat cells were cultured in RPMI1640 medium supplemented with 10% FBS and activated with 10 ng/mL phorbol myristate acetate (PMA; Thermo Fisher Scientific) or with Dynabeads Human T-activator CD3/CD28 (Thermo Fisher Scientific). After activation, the cells were collected, stained with a cell viability dye (Ghost V450, TONOBO), and the following allophycocyanin (APC)-conjugated antibodies: anti-human CD69 (BioLegend, clone FN50), or with isotype control (BioLegend, clone MOPC-173 and clone MOPC-21; isotype 1 and isotype 2, respectively) and analyzed using flow cytometry.

Human peripheral blood mononuclear cells (PBMC) and healthy mouse splenocytes were cultured in RPMI1640 medium supplemented with 10% FBS at a density of 1 × 10^6^ cells/mL. Cells were activated with 25 μL of CD3/CD28 beads per 1 × 10^6^ cells (Gibco, Dynabeads human or mouse T-activator) and recombinant human IL2 (BioLegend). Subsequently, the cells were collected and stained with a cell viability dye (Ghost UV780, TONOBO), blocked with TruStain FcX (BioLegend, human or anti-mouse CD16/32, clone 93), and stained with CD3 (BioLegend, anti-human, clone HIT3a or anti-mouse, clone 17A2) and CD69 (BioLegend, anti-human, clone FN50 or anti-mouse, clone H1.2F3) for flow cytometric analysis.

### CNS and Spleen Tissue Processing

Single-cell suspensions were obtained from spleen and brain tissues, as described previously (47). Briefly, splenocytes were isolated by mechanical disruption of the spleen, passage through a 40-μm filter, and red blood cells (RBC) lysis using ACK lysis buffer (Gibco). Minced tumor tissues were filtered through a 70-μm cell strainer and incubated in a 1X collagenase IV cocktail [1 mL 32 mg/mL (7.4kU) collagenase IV (Worthington, LS004209), 10 mg (53 kU) DNase1 (WorthingtonLS002139), and 20 mg soybean trypsin inhibitor (Worthington LS003587)] for 45 minutes. The samples were then centrifuged for 10 minutes at 2,000 rpm. Cell pellets were suspended in serum-free cell freezing media (Bambanker) at 1 × 10^6^/mL for tumor samples and 3 × 10^7^/mL for spleen samples and were cryopreserved at −80°C until further use.

### Flow Cytometry

Tumor and spleen samples were thawed and resuspended in FACS buffer [Dulbecco PBS (DPBS) + 1% BSA]. For surface staining, cell suspensions were blocked with TruStain FcX (anti-mouse CD16/CD32 BioLegend, clone 93) for 20 minutes on ice and then stained with fluorescently labeled anti-mouse antibodies for 45 minutes in FACS buffer at 4°C. The following antibodies were used: CD45 (BioLegend, clone 30-F11), CD11b (BioLegend, clone M1/70), CD3ε (BD Biosciences, clone 145–2C11), CD8 (BioLegend, clone 53–6.7), CD4 (BioLegend, clone GK1.5), NK1.1 (BioLegend, clone S17016D or PK136), CD69 (BD Biosciences, clone H1.2F3), CD279 (PD-1; BioLegend, clone 29F.1A12), CD366 [T-cell immunoglobulin and mucin domain-containing protein 3 (TIM3); BioLegend, clone RMT3/23], and IgG (BioLegend RTK4530). After staining, cells were washed with FACS buffer and fixed using BD CytoFix/CytoPerm (BD Biosciences). Flow cytometry was performed by LSRFortessa (BD Biosciences). Data were analyzed using FlowJo V10.8.1 data analysis software (FlowJo LLC) and GraphPad Prism v9.2.0.

GL261 cells were cultured in DMEM supplemented with 10% FBS. Cells were subsequently collected and stained with cell viability dye (Ghost V450, TONOBO), IgG (BioLegend, PE anti-mouse IgG2A, κ isotype control), CD69 (eBioscience, APC anti-mouse CD69, clone H1.2F3), and programmed death-ligand 1 [PD-L1 (BioLegend, APC anti-mouse CD274 (B-7-H1, PD-L1), clone 10F.962)], and analyzed by flow cytometry.

### Animals

C57BL/6J female mice, 5–8 weeks old (stock no. 000664), were purchased from the The Jackson Laboratory and housed in the animal facility of the Hillman Cancer Center or UPMC Children's Hospital of Pittsburgh (Pittsburgh, PA). Animals were kept in the facility for at least 1 week before performing any procedures to minimize stress-related symptoms. Female mice were used in all experiments. All experiments were conducted following protocols approved by the University of Pittsburgh Institutional Animal Care and Use Committee (IACUC; approval no. 22101912).

### Intracranial Injections

Murine GL261 cells were cultured as previously described (48). Cells used for intracranial injections were washed twice with DPBS (Lonza), strained through a 70-μm filter, and resuspended in DPBS at 5 × 10^4^ cells/μL. Mice were anesthetized with isoflurane, placed on stereotactic frames (Kopf Instruments), and injected with 1 × 10^5^ GL261 cells in 2 μL of DPBS at coordinates +0.7 mm AP, +2.5 mm ML relative to bregma and −3.0 mm DV using a micropump injector (World Precision Instruments).

### Cell Lines

GL261 cells were obtained from NCI Division of Cancer Treatment and Diagnosis Tumor Repository. Human Jurkat T cells (clone E6–1) were obtained from ATCC. Cells were maintained in liquid nitrogen in Bambanker cryopreservation media until further use. Cells were tested for Mycoplasma using Lonza MycoAlert Mycoplasma Detection Assay Kit prior to any use. Cells were used within 3–5 passages.

### *In Vivo* Reagents

InVivoMAb anti-mouse CD69 (clone CD69.2.2), anti–CTLA-4 (clone 9H10), and anti–PD-1 (clone RPM1–14) were purchased from BioXCell. The deferoxamine (DFO) bifunctional chelator (p-SCN-Bn-DFO) was purchased from Macrocyclics. Zirconium-89 (^89^Zr) was purchased from the Washington University (St. Louis, MO).

### *In Vivo* Treatments

Following injections, mice were divided into two or three different treatment groups (depending on the specific experiment, as detailed in the Results section): ICI treatment, ICI and CD69 blocking, and a DPBS-injected (vehicle) control group. Two ICI treatments were administered on days −3 and 0 in comparison with the tail vein injection and consisted of 200 μg of each ICI (anti–CTLA-4 and anti–PD-1), whereas the control group was injected with 100 μL DPBS per mouse. All injections were of equal volume and were delivered intraperitoneally. On day −1, 500 μg anti-CD69 was injected intraperitoneally into each mouse in the preloaded unlabeled CD69 Ab^+^ ICI-treated group. The experiment was repeated 2–3 times as detailed in the Results section.

### Immunohistology Analysis

GL261 tumors from ICI-treated mice were fixed for 24 hours in neutral phosphate-buffered 10% formalin (Thermo Fisher Scientific) and transferred to 70% ethanol for processing. The tissue samples were dehydrated using a 70% to 100% ethanol gradient over 12 hours, cleared with histologic grade xylene (Leica), embedded in paraffin (Paraplast Plus, Leica), sectioned (4 μm) on Superfrost Plus slides (Thermo Fisher Scientific) using a Leica RM2235 microtome, and baked for 30 or 60 minutes at 60°C. Thereafter, separate staining protocols were used to perform hematoxylin and eosin (H&E) and immunofluorescence (IF) staining.

A Leica ST5020 slide stainer with an integrated cover slipper, CV5030, was used to perform H&E staining with the following staining reagents from Leica: hematoxylin 560MX, blue buffer, aqua define MX, and eosin 515 phloxine. H&E-stained tissue sections were imaged using a Revolve G102 microscope (ECHO) at 1× magnification.

For IF staining, tissue sections were incubated with anti-mouse polyclonal CD69 (AF555, Abcam) and anti-mouse CD3 (AF488, clone 17A2, BioLegend) fluorescence-conjugated antibodies overnight at 4°C. Secondary goat anti-rabbit antibodies were used to detect primary conjugated anti-CD69 antibodies. Tissue sections were additionally stained with Hoechst 33342 (Cell Signaling Technology) to detect cell nuclei following each primary staining incubation as described previously. The stained tissue sections were imaged using a Nikon Ti2-E Eclipse microscope at 40× magnification. NIS Elements software (version blank) was used to analyze the slides for positive staining of CD69 and CD3 antibodies in the tumor tissue. IF Ab staining was performed as described previously with modifications as required (49).

For IHC staining, slides were deparaffinized and rehydrated using a standard histology protocol. Antigen retrieval was performed using citrate buffer (Cell Signaling Technology). The antibodies used were CD69 from GeneTex (GTX37447), and CD3 from Cell Signaling Technology (99940), which were applied using a 1:300 (CD69) or a 1:100 (CD3) dilution at room temperature. The secondary Ab consisted of a Boost Rabbit HRP Polymer (Biocare Medical). The substrate used was 3,3, Diaminobenzidine (Dako), and the slides were counterstained with hematoxylin (Dako).

### MRI

MRI was used to confirm tumor presence and subsequently calculate tumor volume. Mice were anesthetized via a nose cone with 1%–2% isoflurane and O_2_, positioned on an animal bed, and then placed in the scanner. MRI was performed using a 7T/30-cm AVIII spectrometer (Bruker Biospin) equipped with a 12-cm gradient set, an actively decoupled 86-mm quadrature RF volume transmit coil, a 2-channel mouse brain receiver array, and Paravision 6.0.1 software. A T_2_-weighted RARE sequence was used to visualize the tumor, with the following parameters: repetition time (TR)/echo time (TE) = 5,000/40 ms, field of view (FOV) of 20 × 20 mm, acquisition matrix of 128 × 128, 25 slices with a slice thickness of 0.5 mm, four averages, and a RARE factor of 8. DSI Studio (https://dsi-studio.labsolver.org, build: February 20, 2022) was used to manually draw regions of interest (ROI) and calculate the tumor volumes.

### Clone Affinity Assessment

A streptavidin coated 96-well plate (Thermo Fisher Scientific; catalog no. 15520) was washed three times with washing buffer (Tris-buffered saline, 0.05% Tween-20 detergent, 0.1% BSA) and 0.1 μg/100 μL biotinylated mouse CD69 (Creative BioMart) was added to each well. For negative control or nonspecific binding wells, washing buffer was added to wells instead of the biotinylated antigen. The plate was incubated for 2 hours at room temperature with shaking. After washing three times, 100 μL of blocking buffer (Tris-buffered saline, 0.05% Tween-20 detergent, 1% BSA) was added to each well and incubated for 30 minutes at room temperature with shaking. In the next step, all wells were washed with washing buffer, and different concentrations of anti-CD69 [clone CD69.2.2 (BioXcell) or clone H1.2F3 (Invitrogen)] were added to the wells and incubated for 2 hours at room temperature with shaking. Subsequently, all wells were washed three times to remove the unbound antibodies and 0.1 μg/100 μL of secondary Ab [6-His Tag mAb (HIS.H8), horseradish peroxidase (HRP), Invitrogen] was added to each well and incubated for 1 hour and 15 minutes. HRP substrate (HRP Substrate Kit, Bio-Rad) was added to each well and incubated for 15 to 30 minutes and the plate was read on the plate reader (BioTeK) at 415 nm.

### Preparation of ^89^Zr-DFO-CD69 Ab

The conjugation and radiolabeling procedures were performed as previously described, with slight modifications (50). For conjugation, a five-molar-fold excess of p-SCN-Bn-DFO (13.3 μL of 5 mmol/L solution) was added to 1 mL of CD69 Ab (2 mg/mL). The pH was adjusted to 9 by using sodium carbonate (0.1 mol/L). The reaction progress was monitored by size exclusion chromatography-high performance liquid chromatography (SEC-HPLC) using an Agilent 1260 Infinity HPLC (Agilent Technologies) equipped with a Bio SEC-3 4.6 mm x 300 mm column (Agilent Technologies). Using Hamblett method, a DFO substitution number of 1.02 ± 0.26 DFO/Ab was determined (51).

For radiolabeling, we used ^89^Zr, an ideal radioisotope for Ab-based PET imaging studies, because of the consistency between its half-life (*t*_1/2_ = 78 hours), mAb localization, and clearance rates (52–55). A total of 130 MBq ^89^Zr oxalate was used to radiolabel the DFO–CD69 Ab conjugate (1.17 mg/mL). To assess the progress of ^89^Zr labeling, instant thin-layer chromatography was performed using a FlowCount radio-HPLC detection system (Eckert & Ziegler, model 106), then the reaction contents were buffer exchanged to DPBS by PD-10 size exclusion. To determine the radiochemical yield and purity of the conjugated and labeled radiotracers, SEC-HPLC was performed using an Agilent 1260 infinity HPLC (Agilent Technologies) equipped with a Bio SEC-3 4.6 mm × 300 mm column (Agilent Technologies). Specifically, a UV-Vis wavelength of 280 nm was used, which is well-suited for visualizing IgG antibodies (56). First, we ran multiple different IgG antibodies and found that they consistently eluted for approximately 6.7 minutes using an Agilent Bio-SEC 3 column. Successful conjugation without aggregate formation was observed. The gamma radiation elution time of ^89^Zr-DFO-CD69 Ab was 8 minutes, a 1.2-minute delay compared with the elution time of its spectral absorbance, due to the tandem detection of spectral absorbance followed by gamma radiation. The gamma radiation of ^89^Zr-DFO eluted at approximately 10.9 minutes following the spectral absorption of DFO at 9.7 minutes.

### Immunoreactivity

A bead-based radioligand binding assay was performed to assess tracer-binding specificity (57). Recombinant mouse CD69 (R&D Systems) was bound through its Histidine 6-tag to His-tag isolated Ni-NTA Dynabeads (Invitrogen). Nonspecific binding to Ni-NTA was blocked using an IgG Ab with a Histidine-6 tag. These beads were then incubated with ^89^Zr-DFO-CD69 Ab, with or without CD69 Ab (InVivoMAb anti-mouse CD69, clone CD69.2.2). As a control for nonspecific bead binding, ^89^Zr-DFO-CD69 Ab was incubated with Dynabeads and Ni-NTA was blocked with IgG-Histidine-6 Ab. After incubation, the supernatant and beads of each sample were separated and collected to quantify radioactivity in a gamma counter using counts per minute. Triplicates of each experimental condition were used to ensure consistency in the assay results.

### PET/CT and Biodistribution Studies

Immediately after radiotracer production, each mouse was injected intravenously with 100 μL of ^89^Zr-DFO-CD69 Ab (24–46 μg Ab, 1.4–4 MBq ^89^Zr, specific activity: 8.79–13.33 GBq/μmol; a range is depicted since the experiment was repeated). To examine the effect of preloading with unlabeled CD69 Ab, one group was injected intraperitoneally at day −1 in comparison with tail vein injection, with 500 μg of CD69 Ab, reducing the specific activity 10-fold. Subsequently, static immuno-PET imaging was performed using an Inveon Preclinical Imaging Station (Siemens Medical Solutions) at 1, 2, 3, 4, and 6 days posttreatment and tracer tail vein injection. All images were coregistered using the Vivoquant software (version 9), and ROI analysis was guided by CT. Tumor-specific ROIs were generated and presented as the maximum or mean SUVs (SUV_max_ or SUV_mean_). The ROI was also drawn around the left ventricle of the heart at the apex to calculate the blood pool activity as a measure of nonspecific signals, and the SUV_max_ or SUV_mean_ tumor-to-blood ratio (TBR) was calculated. Biodistribution (BioD) studies were performed immediately after the last imaging (day 6) and on days 3 and 8 for ICI-only treated mice. The major organs were collected and weighed, and tissue-associated radioactivity was assessed using a gamma counter as the percent injected dose/gram (%ID/g). The biodistributed samples were counted using a Wizard 2-Detector Gamma Counter (Perkin Elmer, model 2480).

### Single-Cell RNA Sequencing

Two primary GBM scRNA datasets (GSE84465 and GSE131928) were downloaded from the Gene Expression Omnibus (GEO) database (58, 59). One dataset that filtered CD45^+^ cells from primary, recurrent, and post ICI treatment GBM samples (GSE154795) was downloaded from the GEO database (60). Smart-seq2 data were normalized to transcripts per million (TPM), and the normalization of GSE154795 was the same as in the original study (60). Quality control, dimensionality reduction, and cell clustering were performed using Seurat v4. The cell types of the different clusters, annotated using cranial markers, were based on the original study. Visualization of the expression and coexpression of CD69 in different cell types was performed using the Seurat FeaturePlot function and ggplot2 package in Uniform Manifold Approximation and Projection (UMAP) for embedding. Statistical significance was evaluated using ANOVA for the three groups, and pairwise comparisons were evaluated using the Wilcoxon rank-sum test for nonnormally distributed datasets. All the single-cell RNA sequencing (scRNA-seq) datasets we tested had *P* < 2.2e-16 in Shapiro–Wilk test and *P* < 3.7e-24 in Anderson–Darling normality test, denoting that these samples do not have normal distribution. All analyses were performed using R version 4.1.

### Statistical Analysis

Graphics were created using BioRender (https://biorender.com). Statistical analyses, including unpaired Student *t* test and one- or two-way ANOVA, were performed using GraphPad Prism v9.2.0 software. Where appropriate, statistical significance was corrected for multiple comparisons using Tukey method or, for unequal SE, using Welch correction. Kaplan–Meier survival curves were generated to determine survival and then compared using the log-rank Mantel–Cox test. Pearson correlation test was used to determine the relationship between the immuno-PET signals and survival. All data are presented as the mean ± SEM. Statistical significance was determined as indicated in the text and the figure legends. Statistical significance was set at *P* < 0.05. *P* values are as follows: NS, not significant; *, *P* ≤ 0.05; **, *P* ≤ 0.01; ***, *P* ≤ 0.001.

### Data Availability Statement

The scRNA-seq datasets are available through GEO (GSE84465 GSE131928, and GSE154795) and from the authors of these data (58–60).The data analysis generated in this study is available upon request from the corresponding authors.

## Results

### CD69 is Upregulated Upon T-Cell Activation *In Vitro*

To corroborate our hypothesis that CD69 is an ideal imaging agent for monitoring ICI response by virtue of early T-cell activation, we measured CD69 expression on T cells following activation *in vitro* and on GL261 glioma cells. Healthy mouse splenocytes were activated with anti-CD3/CD28 and IL2, and CD69 expression was evaluated at multiple time points postactivation. CD69 expression was negligible preactivation (0 hour) and increased significantly on CD3^+^ T cells at 4 and 24 hours postactivation (3.69% and 32.1%, respectively), followed by a decline in expression at 48 hours (18.96%; Supplementary Fig. S1A).

Similarly, the activation of human PBMCs by anti-CD3/CD28 stimulation resulted in a significant increase in the percentage of CD69^+^ T cells 4 hours after activation (42.4%) and peak CD69 expression at 24 hours (84.2%). Unlike mouse splenocytes, CD69 expression remained high at 48 hours on human T cells (85.1%; Supplementary Fig. S1B). Jurkat cells, an immortalized human T-lymphocyte cell line, activated with PMA, exhibited significant upregulation of CD69 as early as 1 hour postactivation and markedly higher expression of CD69 at 24 hours postactivation (Supplementary Fig. S1C). These results are overall in line with previous published data on CD69 early upregulation upon activation (61, 62), showing early time point of 2 hours with a lasting effect after 48 hours (63).

Because tumor cells, including gliomas, can aberrantly express lineage nonspecific molecules (64), we evaluated CD69 expression on the GL261 glioma cell line (Supplementary Fig. S1D) and dissociated GL261 tumor tissue post ICI treatment by flow cytometry (Supplementary Fig. S1E). As expected, GL261 cells did not express CD69 (MFI = 528.3 vs. isotype MFI = 624.3), but expectedly expressed PD-L1, consistent with the response of GL261 to checkpoint immunotherapy (refs. 47, 65; Supplementary Fig. S1D). Similarly, CD69 was detectable on CD45^+^ hematopoietic cells, but not on CD45^−^ cells from dissociated tumors (Supplementary Fig. S1E). Taken together, CD69, an early activation marker in mouse and human T cells, demonstrates robust expression at 24 hours postactivation and is not expressed in GL261 glioma cells or nonhematopoietic cells within the GL261 tumor microenvironment (TME); suggesting that it can be a suitable biomarker for immuno-PET imaging.

### *In Vivo* Early Activation of CD69 on TILs Following ICI in a GBM Mouse Model

**FIGURE 1:**
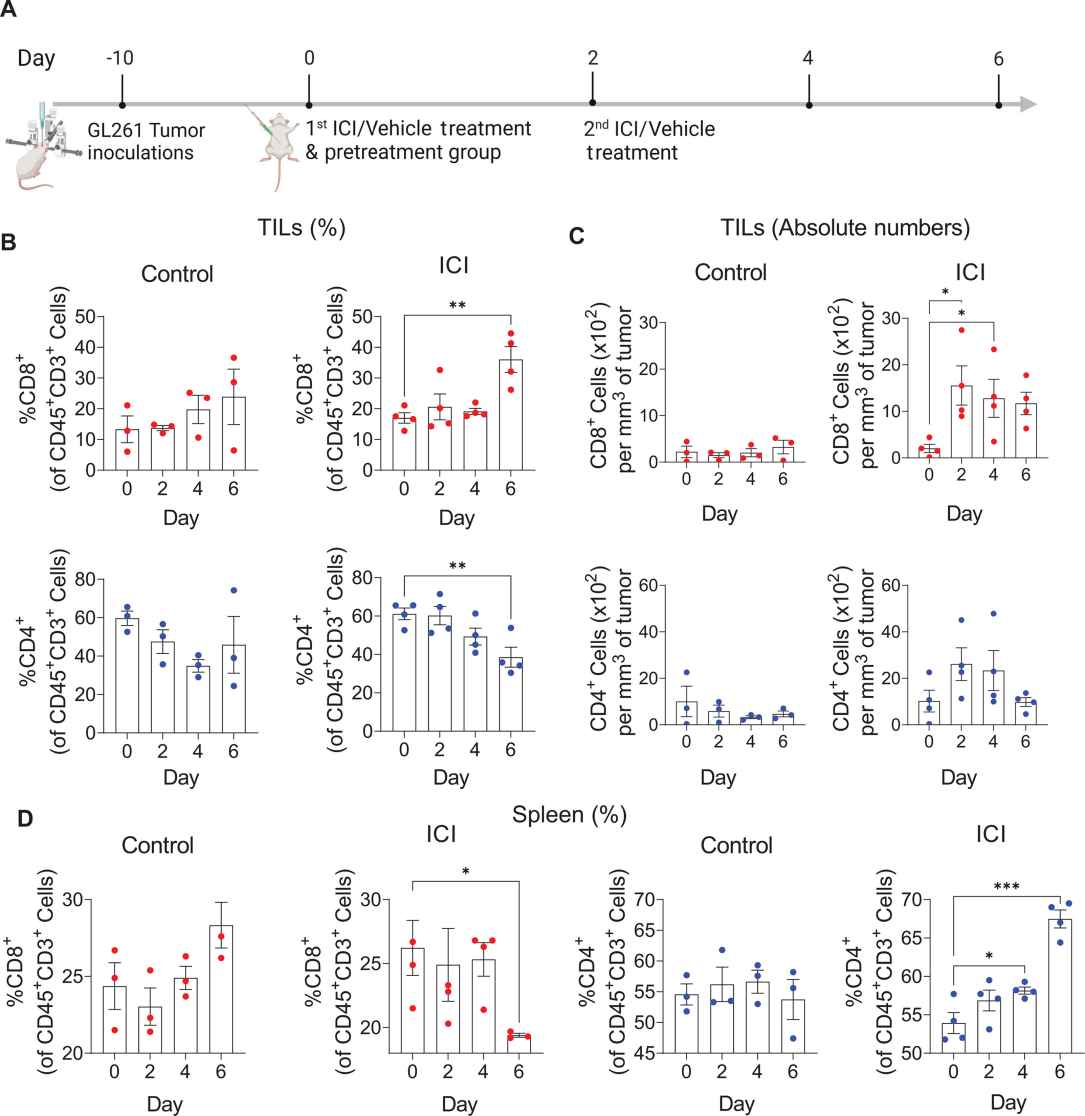
ICIs influence T-cell distribution in tumor and spleen. Mice (*n* = 28) were inoculated with GL261 cells, treated with ICI or vehicle control, and analyzed at the designated time points relative to treatment. **A,** Schematic showing tumor inoculation day, treatment days, and time points relative to treatment in which mice were analyzed. **B,** Percentage of CD8^+^ TILs (red dots) and CD4^+^ TILs (blue dots) in each time point relative to treatment, for vehicle-control group (left panel) and ICI-treated group (right panel) **C,** Absolute number of TILs per mm^3^ of tumor. CD8^+^ cells × 10^2^ (upper panels; red dots) and CD4^+^ cells × 10^2^ (bottom panels; blue dots) in each time point relative to treatment, for ICI-treated group and vehicle-control group. **D,** Frequencies of CD8^+^ (red dots) and CD4^+^ T-cells (blue dots) in spleen in each time point relative to treatment, for vehicle-control group and ICI-treated group. B–D, Shown are means ± SEM. Scattered dots represent individual mice. Shown are representative data of two independent experiments. *n* = 3–4 per group. CD8^+^ and CD4^+^ were gated from CD3^+^CD45^+^ cells. One-way ANOVA with multiple comparison test (*, *P* < 0.05; **, *P* <0.01; ***, *P* <0.001).

To assess the impact of ICI on T-cell accumulation and CD69 expression *in vivo*, mice bearing GL261 GBM tumors were treated with anti–CTLA-4 and anti–PD-1 ICI (200 μg per Ab per mouse; i.p.) or vehicle control on days 10 and 12 after tumor cell inoculation. T-cell populations were examined in dissociated tumors and splenocytes by flow cytometry on days 0 (pretreatment), 2, 4, and 6, relative to treatment (summarized in Fig. 1A). In the ICI-treated group, the proportion of CD8^+^ TILs significantly increased over time while the proportion of CD4^+^ TILs decreased, when compared with pretreatment. Similar trends in T-cell populations were observed in the control group, although not significant (Fig. 1B). We also quantified the absolute number of TILs per cubic-millimeter (mm^3^) of tumor (cell density in tissue) to represent the amount of T-cell accumulation. In the control group, no increase in TILs was observed compared with pretreatment. However, in the ICI-treated group, the number of CD8^+^ TILs increased following ICI treatment (Fig. 1C). CD4^+^ TILs showed a similar trend as CD8^+^ TILs, but was not significant (Fig. 1C). The distribution of T-cell populations in the spleen in the ICI-treated group demonstrated an overall trend of decreased CD8^+^ and increased CD4^+^ T cells over time, which reached statistical significance when compared with pretreatment. This trend was not found in the control group, where CD4^+^ T cells kept an overall stable percentage over time, and CD8^+^ T cells showed a trend of increase in proportion over time that did not reach any significance (Fig. 1D). Overall, these data demonstrate elevated CD8^+^ TILs and their decreased proportions in the periphery upon ICI treatment (Fig. 1B–D). These findings are in line with previous reports of high intratumoral, but not circulating, CD8^+^ T cells as predictors of ICI treatment outcomes (66).

**FIGURE 2:**
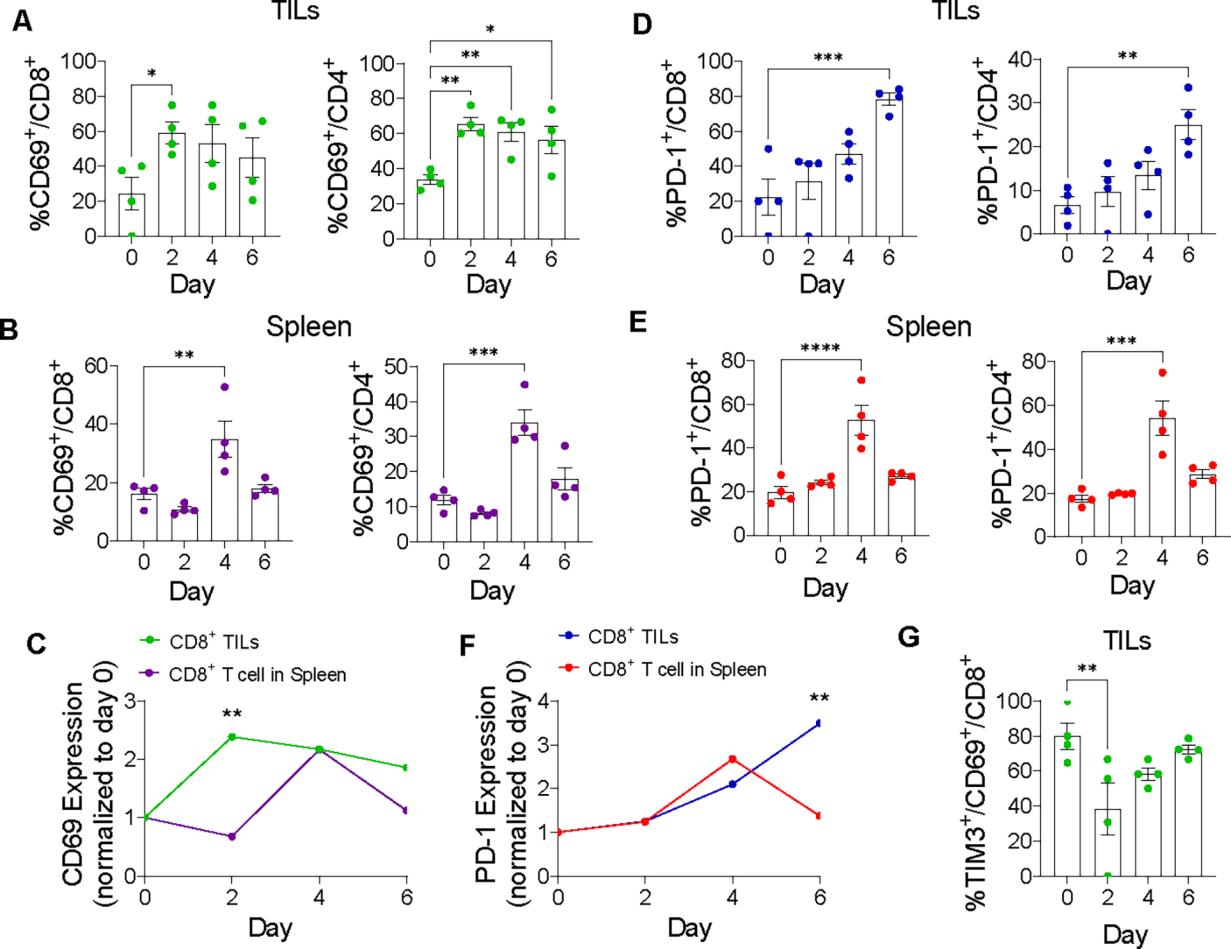
Kinetics of activation markers on T cells following ICI treatment. Mice with intracranial GL261 gliomas were treated with ICI, and analyzed at the designated time points after treatment, as in Fig. 1. **A,** Percentages of CD69^+^ of CD8^+^ (left) and CD4^+^ TILs (right). **B,** Percentages of CD69^+^ of CD8^+^ (left) and CD4^+^ T cells (right) in spleen. **C,** Trajectories of CD69^+^/CD8^+^ in TILs (green line) and spleen (purple line) ratios at the designated time point compared with the pretreatment group. **D,** Percentages of PD-1^+^ of CD8^+^ (left) and CD4^+^ TILs (right). **E,** Percentages of PD-1^+^ of CD8^+^ (left) and CD4^+^ T cells (right) in spleen. **F,** Trajectories of PD-1^+^/CD8^+^ in TILs (blue line) and spleen (red line) ratios at the designated time point compared with the pretreatment group. **G,** Percentages of TIM3^+^ of CD69^+^/CD8^+^TILs. For A, B, D, E, and G, mean ± SEM values are shown. Scattered dots represent individual mice. Shown are representative data of two independent experiments. *n* = 4 per group. One-way ANOVA with multiple comparison test (*, *P* < 0.05; **, *P* <0.01; ***, *P* <0.001). C and F, Two-way ANOVA with multiple comparison test is used to compare the ratios between TILs and spleen T cells at each time point (*, *P* < 0.05; **, *P* <0.01; ***, *P* <0.001).

We then investigated the kinetics of CD69 expression on TILs and splenic T cells after ICI treatment in relation to PD-1 expression, a marker previously evaluated as an immuno-PET target on TILs (ref. 67; Fig. 2). CD69^+^ expression was significantly increased on CD8^+^ and CD4^+^ T cells both within the tumor (Fig. 2A) and spleen (Fig. 2B). Notably, a significant increase in the expression of CD69^+^ on TILs was observed at an earlier timepoint (2 days) compared with that in splenic T cells (4 days). Moreover, on day 2, there was a significant difference between CD69 expression on TILs and splenic T cells, as shown by the ratio of post/pretreatment CD69^+^/CD8^+^ T cells (Fig. 2C). Similar kinetics of PD-1 and CD69 were observed in splenic T cells (a significant increase in 4 days; Fig. 2B and E); however, upregulation of PD-1^+^ on TILs occurred at a later timepoint (6 days; Fig. 2D and F), compared with CD69 (2 days; Fig. 2A and C). As with CD69, PD-1 expression on TILs at the mentioned timepoint (6 days) was significantly higher than that on splenic T cells (Fig. 2F). Together, these data demonstrate that CD69 is an early T-cell activation marker specifically in the TME after ICl, demonstrating its potential for early immuno-PET monitoring of ICI response.

Given that some exhausted T cells also express CD69 (68, 69), we evaluated the T-cell marker of exhaustion TIM3 on CD69-expressing CD8^+^ TILs upon ICI treatment (70, 71). While TIM3 was expressed on approximately 80% of CD69^+^/CD8^+^ TILs prior to ICI, 2 days post ICI, the frequency of TIM3 on CD69^+^CD8^+^ TILs deceased significantly (<40%). Thereafter, the percentage of TIM3^+^/CD69^+^/CD8^+^ trended toward pretreatment levels (Fig. 2G). This supports the finding that ICI treatment leads to increased activation of CD8^+^ TILs.

### CD69 within GBM is Predominantly Expressed on Lymphocytes and Increases Following ICI Treatment in a Mouse Model and in Patients

To further validate the expression of CD69 on TILs, we visualized orthotopic GL261 tumors from ICI-treated mice using microscopy following H&E, IF, and IHC staining. H&E staining confirmed the presence of tumors and showed histologic characteristics of GBM (72), including infiltrative growth, nuclear anaplasia, mitotic activity, endothelial proliferation, and necrosis (Supplementary Fig. S2A). Consistent with the flow cytometry results, lymphocytes were readily observed infiltrating the tumors (TIL) within the H&E-stained sections (Supplementary Fig. S2B). IF staining of tumor and spleen tissues from ICI-treated animals demonstrated the expression of CD69 on T cells via the coexpression of CD69 and CD3, putatively representing activated T cells (Supplementary Fig. S2C and S2D, respectively). In addition, we performed IHC staining to evaluate the expression of CD69 on TILs from GL261 tumor–bearing mice in control and ICI-treated animals. As positive control staining, we evaluated IHC on spleen tissue for CD3 and CD69 (Supplementary Fig. S2E). In the ICI-treated group, TILs were identified in different areas with CD3 expression, and CD69 staining on serial section slides suggested coexpression of CD69 on TILs (Supplementary Fig. S2F). Control mice exhibited clear CD3 staining without corresponding CD69 expression (CD69 had background nonspecific staining; Supplementary Fig. S2G). Together, these data support the expression of CD69 on CD3^+^ T cells in the tumor and spleen tissues of GBM-bearing mice following ICI treatment and further support its use as a putative biomarker of immunotherapy response.

Next, we examined the potential of CD69 as a biomarker of ICI response in patients with GBM. scRNA-seq data were analyzed from two published datasets of primary IDH-wild-type GBM (58, 59). The first dataset comprised a cohort of four primary GBM adult patients (3,589 cells in total), and the other combined scRNA-seq of 20 adults and 8 pediatric patients with GBM (24,131 cells in total). We first examined CD69 expression across different cell types and found that it was expressed almost exclusively on immune cells and not on tumor cells or CNS resident cells (Supplementary Fig. S3A and S3B). This is in line with our data showing no CD69 expression on GL261 cells or nonhematopoietic cells from dissociated GL261 tumors (Supplementary Fig. S1). We then examined different immune cell types from scRNA-seq data and found that CD69 expression was significantly higher on T cells than macrophages (Supplementary Fig. S3C), supporting the notion that CD69 is a prominent marker of T-cell activation.

**FIGURE 3:**
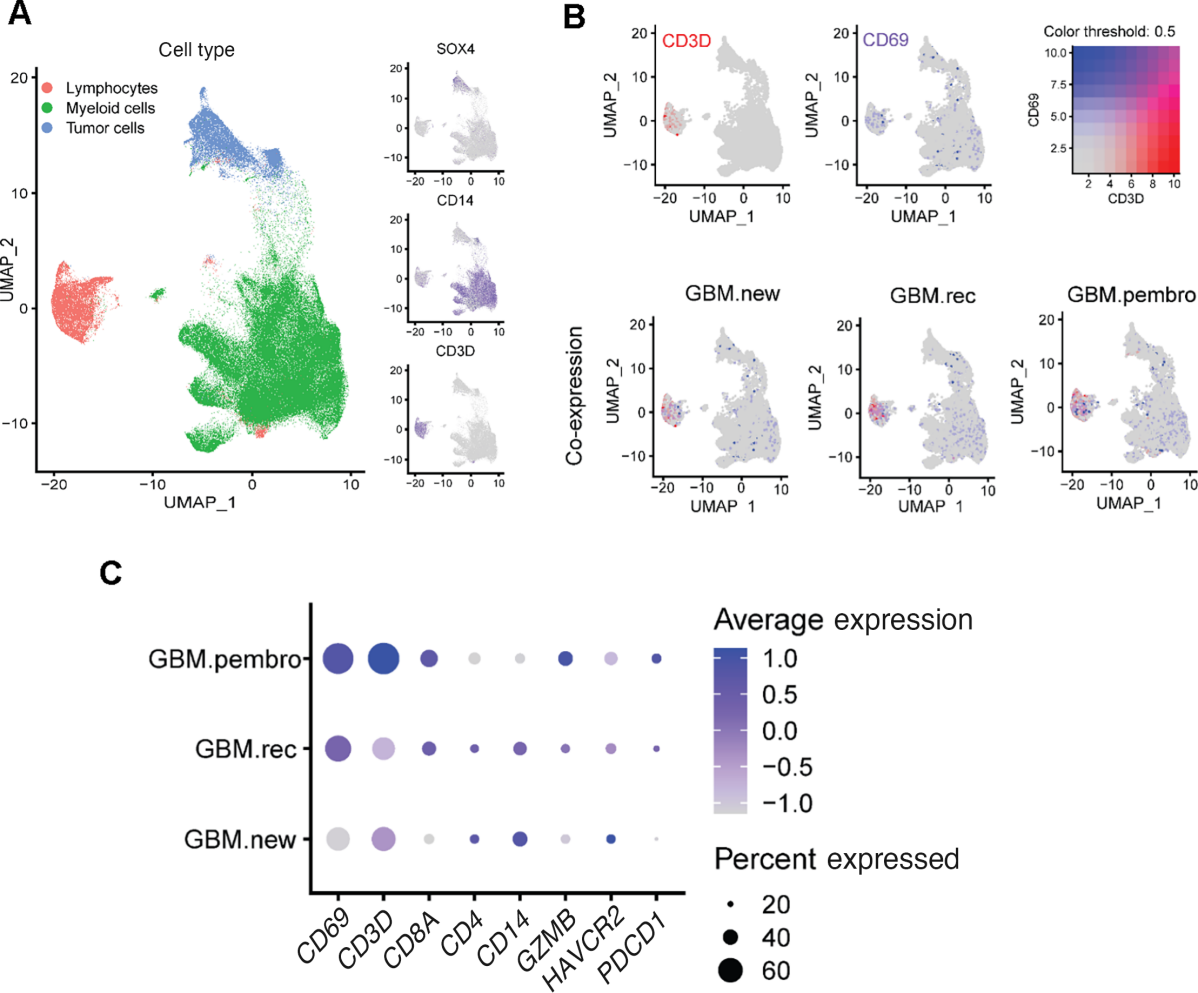
CD69 expression increases on TILs following ICI treatment in patients with GBM. **A–C,** scRNA-seq analysis of sorted CD45^+^ immune cells (*n*  =  156,766 cells) from 40 patients with GBM: 14 GBM.new, 12 GBM.rec, and 14 GBM.pembro. **A,** UMAP cell clustering analysis of tumor cell cluster (SOX4; top right), myeloid cell cluster (CD14; middle right), and lymphocyte cluster (CD3D; bottom right). **B,** UMAP data color-coded by the expression of CD3 (red), CD69 (purple; top), and coexpression (merge) in each group of patients (bottom). **C,** Dot plot analysis of cells within the lymphocyte cluster; the size of the dots corresponds to the percentage of cells expressing the feature (gene). The color represents the average expression level.

We next examined the influence of ICI treatment on CD69 expression, and the ability of this molecule to serve as an immunotherapy response marker relevant to patient imaging. To do this, we used a scRNA-seq dataset that examined the impact of neoadjuvant anti–PD-1 (neo-aPD-1) on the immune landscape of the TME. The dataset included tumor-infiltrating CD45^+^ immune cells isolated from 40 patients: 14 newly diagnosed GBM (GBM.new), 12 recurrent GBM without prior immunotherapy (GBM.rec), and 14 recurrent GBM with neo-aPD-1 therapy (GBM.pembro; *n*  =  156,766 cells in total; ref. 60). CD69 was predominantly expressed on CD3^+^ lymphocytes and, to a lesser extent, on myeloid/microglia cells, with an apparent increase following neo-aPD-1 treatment (Fig. 3A and B; Supplementary Fig. S3D). Analysis of the scRNA-seq lymphocyte cluster showed the highest amounts of CD69, CD3, CD8 in pembro.GBM compared with the “new” and “recurrent” GBM groups (Fig. 3C). Also, the pembro.GBM group had the highest expression of granzyme-B (*GZMB*) and PD-1 (*PDCD1*) genes and lowest expression of CD4 and TIM3 (*HAVCR2*) genes (Fig. 3C). We further investigated T-cell subpopulations from the scRNA-seq data and found significant increase of CD69 expression across cell types including on early activated T cells, effector CD8^+^, and effector progenitor T cells in the cohort of GBM.pembro compared with the other two cohorts (Supplementary Fig. S3E). These T-cell subsets are known to contribute to antitumor immunity (73–75). Of note, CD69 expression was increased following ICI treatment on T-regulatory cells, but only when compared to GBM.new patients but not GBM.rec (Supplementary Fig. S3E). Together, these data support that CD69 is a putative biomarker for monitoring changes of T-cell immune responses in the TME following ICI administration in patients with GBM.

### Immuno-PET of ^89^Zr-DFO-CD69 Ab Visualizes the TME after ICI Treatment in a GBM Mouse Model

To noninvasively quantify CD69 expression *in vivo*, as a marker of ICI response, we generated a CD69 immuno-PET imaging agent, ^89^Zr-DFO-CD69 Ab.^89^Zr-DFO-CD69 Ab was produced by a two-step synthesis reaction, starting with conjugation of CD69 Ab to DFO, yielding a median molar ratio of one DFO/Ab, followed by radiolabeling of DFO-CD69 Ab with ^89^Zr (Supplementary Fig. S4A; refs. 48, 50). The anti-CD69 clone (CD69.2.2) used has been widely published and is known to react with mouse CD69 *in vivo* without depleting CD69-expressing cells or interfering with T-cell priming (76–80). SEC-HPLC and gamma radiation detection each showed a single peak, indicative of successful conjugation and radiolabeling (Supplementary Fig. S4B and Methods). We then determined the binding fraction of the radiolabeled conjugate by immunoreactivity analysis. The radiotracer-bound CD69 antigen with high immunoreactivity (93.9% ± 0.2%), while preloading with unlabeled CD69 Ab (18% ± 1.4%), and control (12.1% ± 1.3%) groups were significantly lower, indicating the functionality and high immunoreactivity of our radiotracer (Supplementary Fig. S4C and Methods). Of note, we performed affinity comparison studies comparing two anti-CD69 Ab clones; H1.2F3 and CD69.2.2 (Supplementary Fig. S5). Both of the clones demonstrated high affinity to mouse CD69 (*K*_d_ < 20 nmol/L); clone CD69.2.2 had a binding affinity of 14 ± 4 nmol/L while clone H1.2F3 demonstrated 3.9 ± 2 nmol/L with insignificant differences between the affinity binding (*P* = 0.9). Therefore, while both clones are suitable for immuno-PET studies, we continued to investigate clone CD69.2.2 due to its known reactivity with mouse CD69 *in vivo* without depleting CD69-expressing cells or interfering with T-cell priming (77, 80), and its well established use in the field (76–80).

**FIGURE 4:**
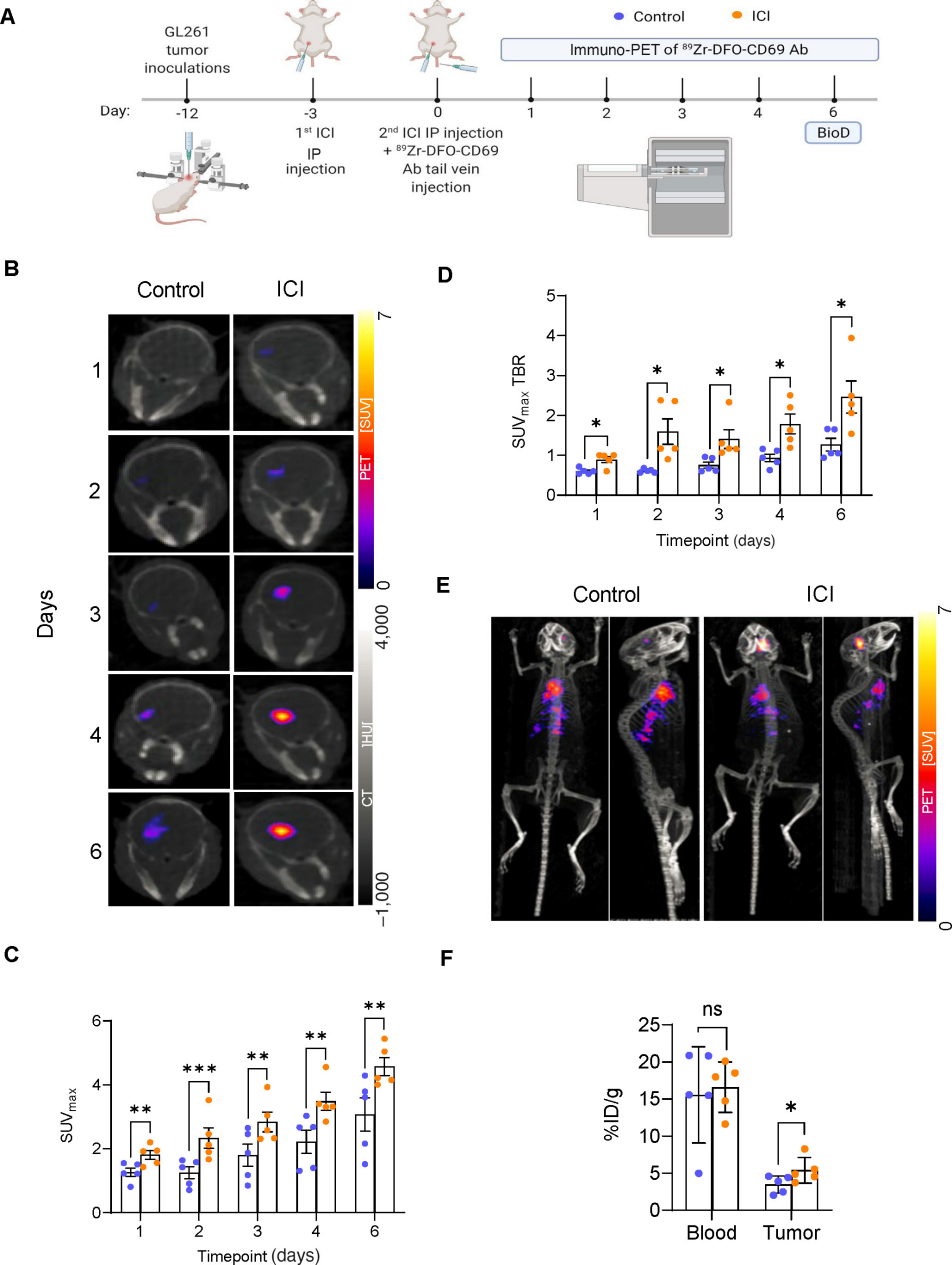
Immuno-PET of ^89^Zr-DFO-CD69 Ab visualizes the TME after ICI treatment in a GBM mouse model. Mice were inoculated with GL261 cells, and evaluated by immuno-PET of ^89^Zr-DFO-CD69 Ab, comparing the treatment (ICI) group to the control group (represented by orange and blue dots, respectively). **A,** Schematic showing timeline of tumor inoculation, ICI treatment, tail vein injection of ^89^Zr-DFO-CD69 Ab, immuno-PET, and BioD. **B,** Representative coronal head images from immuno-PET of ^89^Zr-DFO-CD69 Ab of ICI-treated and control mice at designated time points. Scales show SUVs of PET (SUV; colored) and CT (HU; gray). **C** and **D,** Comparison between ICI-treated mice and control at designated time points of tumor-specific regions’ SUV_max_ (C) and SUV_max_ TBR (D). **E,** Representative coronal-ventral and sagittal 3D whole-body MIP PET images of ^89^Zr-DFO-CD69 Ab of control and ICI-treated mice acquired 6 days after tracer administration. The scale shows SUVs of PET. **F,** Day 6 BioD results of blood- and tumor site–associated radioactivity, assessed as %ID/g. In C, D, and F, bars show means ± SEM. Scattered dots represent individual mice. Shown are representative data of three independent experiments, *n* = 5 per group. C and D, Multiple unpaired *t* test with Welch correction. F, Two-way ANOVA with multiple comparison test (*, *P* < 0.05; **, *P* <0.01; ***, *P* <0.001). IP, intraperitoneal.

To evaluate ^89^Zr-DFO-CD69 Ab for *in vivo* immuno-PET, mice with established GL261 tumors treated with ICI or vehicle control received tail vein injection of ^89^Zr-DFO-CD69 Ab. Subsequently, static PET/CT imaging was performed 1, 2, 3, 4, and 6 days posttreatment and tracer injection. BioD analysis was performed after the final immuno-PET scan on day 6 (summarized in Fig. 4A).

We performed a semiquantitative analysis of the tracer signal by determining the SUV_max_ of the tumor-specific regions. At every time point assessed, immuno-PET scans showed a significantly higher SUV_max_ in the tumors of ICI-treated mice than in the control group. Furthermore, in each group, CD69 expression increased over time (Fig. 4B and C). Notably, the most significant difference between the groups was observed on day 2. These data confirm that CD69, a marker for T-cell activation, can be detected by immuno-PET and is significantly higher after ICI treatment, with an increase in tracer SUV over time.

Next, specific ^89^Zr-DFO-CD69 Ab PET imaging uptake was calculated by dividing the tumor uptake by left heart ventricle uptake to derive the TBR, a previously reported measure of tumor-retained signals (81). An SUV_max_ TBR >1 putatively represents refined tumor-specific uptake that controls nonspecific tracer signals from the blood. Tumor-specific tracer uptake was significantly higher in the ICI-treated group than in the control group at all time points. In addition, in the ICI-treated group, tumor-specific uptake was observed starting on day 2 and increased over time. Strikingly, on day 6, we noted very specific tumor uptake, as indicated by SUV_max_ TBR >2. However, in the control group, tumor-specific uptake was noted only on day 6 and was only slightly higher than that in the blood (SUV_max_ TBR mean = 1.27; Fig. 4D). Evaluation of SUV_mean_ values and SUV_mean_ TBRs demonstrated similar results, although less striking, compared with our SUV_max_ analysis (Supplementary Fig. S6A). Notably, SUV_max_ values generated following manual tumor segmentation provide higher interobserver precision than SUV_mean_ values as the former is not influenced by mild differences in segmentation. These results suggest that our tracer uptake depicts a unique ICI-induced immune process occurring at the tumor site and not only a representation of the blood-containing tracer within the tumor due to a disturbance of the blood–brain barrier.

On day 6, the maximum intensity projection (MIP) 3D method was used to visualize the distribution of the tracer throughout the body. In ICI-treated mice, we observed higher MIP SUV signals at the tumor site compared with the systemic tracer localization found in the chest cavity and upper gastrointestinal system. In our control group, we detected a slightly higher MIP SUV signal at the same general systemic locations, but a much weaker MIP signal at the tumor site (Fig. 4E). These results support that the transition of the tumor from “cold” to “hot” is acquired through increased immune cell activation that is tumor specific as a result of the ICI treatment and not solely a derivative of the blood-contained tracer.

BioD studies performed immediately after the last imaging (day 6) were used to evaluate tissue-associated radioactivity as %ID/g. A significantly higher accumulation of our tracer was found in the tumors of the ICI-treated group than in those of the control group, whereas radioactivity levels in the blood were quite similar between the groups (Fig. 4F). This validates our immuno-PET SUV imaging results and further supports tumor-specific tracer uptake of ^89^Zr-DFO-CD69 Ab following ICI treatment. Tracer accumulation in the spleen and thymus appeared to be higher in the ICI-treated group; however, these differences were not significant. No significant differences were observed in other organs (Supplementary Fig. S6E).

To further validate the tumor accumulation of our tracer over time, we examined the BioD of ^89^Zr-DFO-CD69 Ab on days 3 and 8 after ICI treatment (Supplementary Fig. S6B). We observed a significant decrease in the tracer in the blood over time, simultaneously with a significant increase in tracer uptake at the tumor site (Supplementary Fig. S6C). These data suggest tumor-specific uptake of the tracer, which is not derived solely from the blood tracer content. In addition, we observed an increase in the tracer in the spleen, which is known to have a high lymphocyte content, further supporting the T-cell–specific uptake of the tracer (Supplementary Fig. S6C). Competitive inhibition with CD69 Ab eliminated tracer binding to CD69 *in vitro*, demonstrating the high specificity of ^89^Zr-DFO-CD69 Ab (Supplementary Fig. S4C). *In vivo*, preloading with unlabeled CD69 Ab administered 24 hours prior to tracer injection significantly reduced tracer uptake in the immune periphery (i.e., spleen) while enhanced tumor-specific ^89^Zr-DFO-CD69 Ab uptake, as indicated by the SUV values (Supplementary Fig. S6D) and BioD data (Supplementary Fig. S6E). This is in accordance with literature showing that preloading with unlabeled antibodies can improve site-specific PET/CT imaging by competitively inhibiting systemic tracer uptake (82).

Of note, when evaluating an immuno-PET agent with antigen expressed in tissues other than the tumor, on-target off-site “antigen sink” captures radiotracer resulting in decreased tumor accumulations (83, 84). Therefore, *in vivo*, an isotype control immuno-PET radiotracer will circulate in the blood without getting captured and have noncomparable readouts for our CD69 radiotracer. Thus, a ^89^Zr-labeled isotype-matched antibody serves as a poor control for nonreceptor-mediated target tissue accumulation. In addition, to determine the potential for Fc region–mediated radiotracer accumulation, we examined nonspecific staining of *in vitro*–activated T cells and immune cells from dissociated tumor tissue following ICI treatment and did not detect binding of isotype control antibody, with the MFI of the isotype controls being similar to the unstained cells (Supplementary Fig. S7A and S7B). These data suggest that the accumulation within the TME is not Fc mediated.

### Immuno-PET of ^89^Zr-DFO-CD69 Ab as a Prognostic Predictor after ICI Treatment in a GBM Mouse Model

**FIGURE 5:**
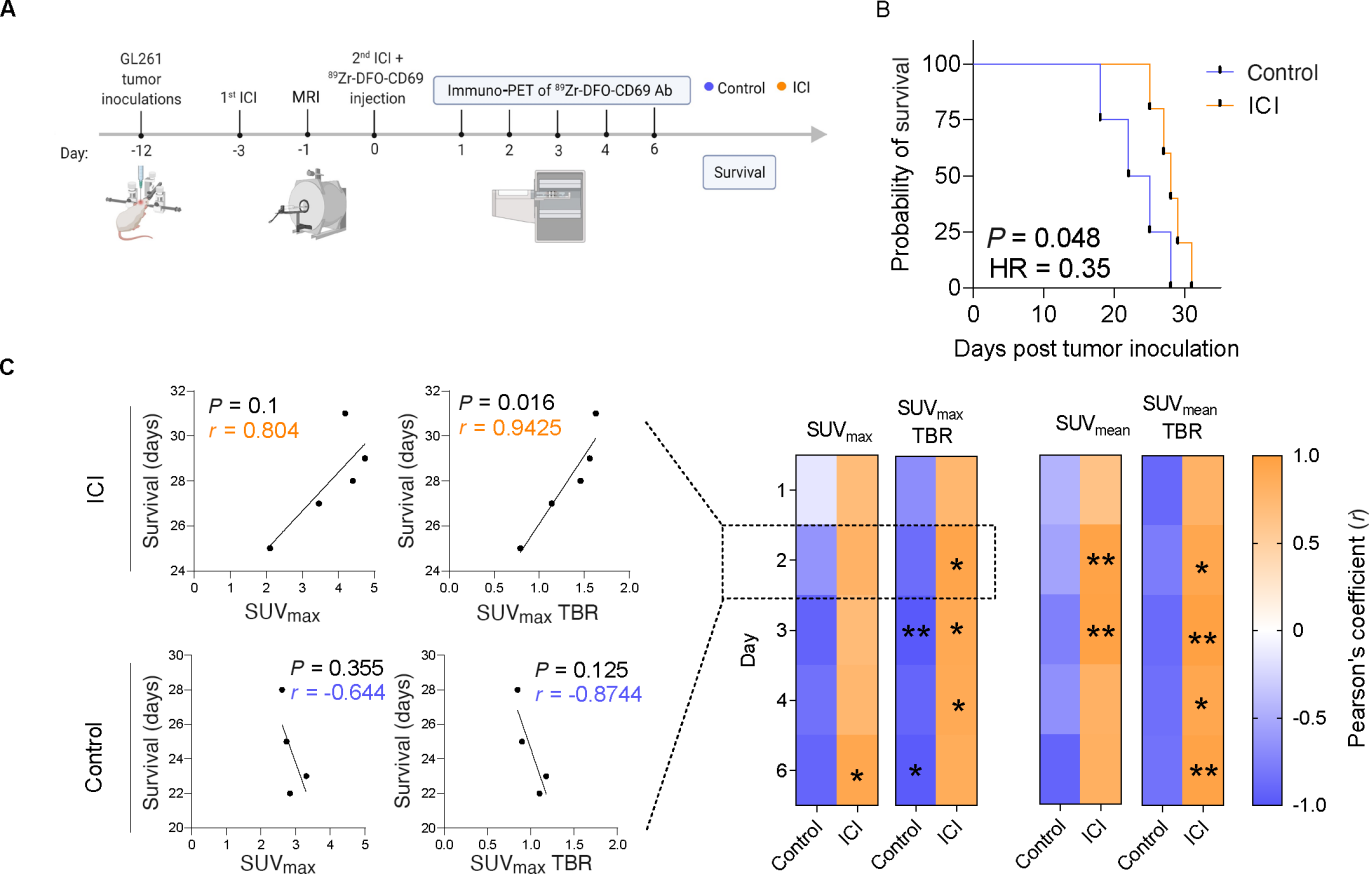
Immuno-PET of ^89^Zr-DFO-CD69 Ab as a prognostic predictor after ICI treatment in a GBM mouse model. Survival follow-up of mice inoculated with GL261 cells, and evaluated by immuno-PET of ^89^Zr-DFO-CD69 Ab, comparing treatment (ICI) group to control group (represented by orange and blue dots, respectively). **A,** Schematic showing timeline of tumor inoculation, ICI treatment, MRI, tail vein injection of ^89^Zr-DFO-CD69 Ab, immuno-PET, and survival follow-up. **B,** The overall survival (OS) rates of the ICI-treated group and control group are plotted using Kaplan–Meier survival curves, with log-rank (Mantel–Cox) curve comparison test. **C,** Right, heat map display of Pearson correlation coefficient analysis between immuno-PET signals and survival in the two groups. Results are shown for each SUV measurement method (SUV_max_, SUV_max_ TBR, SUV_mean_, and SUV_mean_ TBR) in each immuno-PET designated time point (days 1, 2, 3, 4, and 6). Shown are representative data of two independent experiments, *n* = 4–5 mice per group (*, *P* < 0.05; **, *P* <0.01). Left, examples of scatter diagrams of Pearson correlation coefficient taken from immuno-PETs on day 2 of SUV_max_ and SUV_max_ TBR. Scattered dots represent individual mice, *n* = 4–5 mice per group.

Our data identified CD69 as an immuno-PET biomarker of T-cell activation upon ICI immunotherapy in a GBM model. Because T-cell activation, measured by CD69 upregulation, is key for successful immunotherapy, durable response, and favorable outcome, we tested whether immuno-PET of ^89^Zr-DFO CD69 Ab correlates with survival after ICI treatment in our model (summarized in Fig. 5A). MRI data showed that the overall tumor volume and volume distribution were equivalent between the groups the day prior to tracer injection. A slightly higher tumor volume, albeit nonsignificant, was observed in the ICI-treated group, with a mean of 5.74 mm^3^ versus 4.86 mm^3^ in the control group (Supplementary Fig. S8). The overall survival (OS) was significantly higher in the ICI-treated group, with a mean of 28 days posttumor inoculation compared with 23.2 days in the control group, with HR showing a 35% decrease in mortality in those treated (Fig. 5B).

Correlation analysis (Fig. 5C; Supplementary Fig. S9) between SUV measurements (SUV_max_, SUV_mean_, SUV_max_ TBR, and SUV_mean_ TBR) and survival (i.e., prognosis) with ICI immunotherapy showed a strong positive correlation (*r* > 0.7) across all immuno-PET time points; higher SUV values, representing T-cell activity, matched longer OS. Even though SUV_mean_ and SUV_mean_ TBRs showed better and more striking results at most time points compared with our SUV_max_ analysis, we prefer to address the SUV_max_ and SUV_max_ TBR results, as we have noted before, as they are more consistent and provide higher interobserver reliability. Hence, when focusing on SUV_max_ analysis, a notable statistically significant correlation was observed on SUV_max_ TBR day 2 after ICI treatment (*r* = 0.9425, *P* = 0.016).

While all SUV measurements correlated with the clinical outcome of mice in both groups, we observed inverted results (i.e., a negative correlation) between the SUV measurements and OS in the control group (not treated with ICI), reflecting that higher SUV values correlated with a worse prognosis. It is important to note that the negative correlation was weaker than that in the ICI group, reflected by frequent results of *r* < −0.5 and a wider range (*r* = −0.15 to −0.99). As in the ICI group, the most notable statistically significant correlation in the control group was observed in SUV_max_ TBR; however, this occurred on day 3 (*r* = 0.9937, *P* = 0.0063) compared with day 2 in the treated group (Fig. 5C; Supplementary Fig. S9).

To investigate the role and strength of our functional CD69 immuno-PET imaging, we assessed anatomic information obtained by longitudinal MRI at the same time frame as the immuno-PET in both ICI treatment and control group. Unlike the statistically significant changes observed from immuno-PET CD69 signal, tumor sizes obtained by MRI were not significantly different between the groups at these timepoints, despite some mild reduction in tumor size for the ICI-treated group on day 6 (Supplementary Fig. S10).

These data show that immuno-PET of ^89^Zr-DFO-anti-CD69 not only correlates with prognosis upon ICI immunotherapy and is therefore a potential imaging tool for immunotherapy treatment guidance but may also serve as an imaging tool for prognosis prediction in nontreated patients, especially when using SUV_max_ TBR measurements on days 2 and 3, respectively.

## Discussion

GBM is an aggressive brain tumor with limited therapeutic options (1, 2), which has motivated the investigation of more effective therapies. Immunotherapy represents a significant advance in the treatment of many cancers and may hold promise for GBM treatment. However, oncologists must balance the benefits that are eventually observed in a small group of patients with immune-related adverse events (85).

Standard clinical imaging modalities, typically used to monitor therapeutic efficacy, have shown limited success in immunotherapy. This is partly due to the apparent radiologic tumor progression from treatment-induced inflammatory infiltrates (86), which is difficult to distinguish from true tumor progression (87, 88). Therapeutic response assessments are currently based on presumed tumor progression rather than on earlier predictive biomarkers of response to therapy. Therefore, alternative imaging methods and biomarkers for monitoring early antitumor immune responses are urgently needed. To date, several successful methods for visualizing specific immune markers have been developed for systemic cancers (33, 89–91), with limited success for GBM, posing greater challenges for immuno-PET monitoring (34). In this study, we investigated the utility of CD69 immuno-PET for molecular imaging to predict therapeutic responses to immunotherapy for GBM.

CD69 is a transmembrane protein that is predominantly expressed on T cells, with some expression on other immune cells, including NK cells (35, 38, 92). CD69 is rapidly upregulated on T cells upon productive T-cell receptor (TCR) engagement with cognate antigens (62), and thus can be a promising biomarker of T-cell activation for early immunotherapy response monitoring. Recent work on CD69 immuno-PET in a murine colon carcinoma model demonstrated an association between tracer uptake and response to ICI (46), further supporting the use of CD69 immuno-PET for immunotherapy response assessment in GBM.

Here, we demonstrate that CD69 can be detected by PET in the GBM TME to monitor ICI treatment response. Our immuno-PET of ^89^Zr-DFO-CD69 Ab in a murine GBM model enabled repeated, quantitative, and noninvasive PET imaging assessments. We found significantly higher tracer uptake in the tumor area of ICI-treated GBM mice than in vehicle-treated mice. In addition, and important for clinical translation, we showed a strong positive correlation between SUV and survival after ICI immunotherapy. Interestingly, our data on high TBR using PET and BioD demonstrated a specific immune activation process occurring within the tumor site.

Our *in vitro* and *in vivo* studies consistently pointed to 48 hours post ICI treatment as the time point for high CD69 signal expression, and a strong association with survival. If future studies show similar findings, this early time point could have important implications for clinical practice. For instance, patients can be administered intravenous with both ICI and the tracer on the same day and then back for only one immuno-PET imaging two days later, allowing the process to be efficient for the early detection of responses.

While we observed a strong positive correlation between survival and SUV in the ICI group, we observed a negative correlation between SUV measurements and OS in the control group (higher SUV values correlated with worse prognosis). These data show that immuno-PET of ^89^Zr-DFO-CD69 Ab may also serve as an imaging tool for prognosis prediction in patients outside the context of immunotherapy. Interestingly, tumor sizes from MRI data on the day prior to tracer injection, between doses on ICI, did not correlate with either survival or CD69 SUV signals in the ICI-treated group, and MRI on day 6 could not provide evidence of difference between the treatment groups. Overall, these data suggest that CD69 immuno-PET is able to detect changes in the tumor immunobiology prior to changes in tumor size, strengthening its potentially useful as a predictive biomarker of ICI responses.

Consistent with our mouse data, our analysis of scRNA-seq data obtained from specimens of patients with recurrent GBM treated with neoadjuvant anti–PD-1 and control cohorts revealed that CD69 expression, along with other effector markers, was significantly elevated on TILs from ICI-treated patients compared with the control group. This finding strengthens the rationale for its translation into clinical practice. We hope, future studies would assess the utility of CD69 immuno-PET in predicting responses in clinical trials and routine clinical work to distinguish treated responders from non-responders. Non-responders could avoid the often-severe side effects of ineffective costly therapy and would have an opportunity to try other, potentially more effective regimens, immunotherapy-based, or otherwise, at an earlier time point.

This study has several limitations. In this study, we used the GL261 model to assess our tracer for monitoring immunotherapy responses in GBM. While GL261 has major benefits as a well-established orthotopic syngeneic model, which allows for evaluation of immunotherapies (93, 94), one of the limitations of GL261 is that its high mutational burden may not represent the majority of patients with GBM (94, 95). Future studies validating CD69 immuno-PET in genetically engineered or *de novo* mouse GBM models can therefore improve relevance for a heterogenous population of patients with GBM. In addition, while our study was performed under “sterile” conditions, in which no external or internal immune challenges are introduced besides immunotherapy, patients usually receive ICI following or in parallel to other treatments. One example is corticosteroids, which are often prescribed to decrease local edema and suppress the immune response, which might affect the immuno-PET results (96). Another example is intercurrent infection, which is commonly introduced during the disease process, and its effects on immune activation. Therefore, future studies should evaluate the effects of “real-world” events on imaging. In addition, we assessed CD69 immuno-PET only for short-term ICI immunotherapy; however, this strategy may be informative for monitoring long-term ICI treatment and other T cell–mediated immunotherapies. Therefore, the validation of CD69 immuno-PET imaging for these scenarios is warranted in future studies. Finally, this study did not assess the biologic impact of antibody binding to CD69, which although given in small doses, may influence T-cell activity. Previous work has shown that anti-CD69 Ab can enhance T-cell antitumor activity in systemic cancers (68, 77, 78). While future studies should assess the dose effects of anti-CD69 administration in GBM, we anticipate that optimization of molar activity can reduce the amount of the anti-CD69 Ab that would be administered.

Overall, we demonstrated that CD69 on TILs represents a promising biomarker for early detection of response to ICI therapy in GBM. Preclinical CD69 immuno-PET using ^89^Zr-DFO-CD69 Ab was found to be highly sensitive for the detection of the anti-GBM immune response and served as a predictor of prognosis upon ICI therapy. Furthermore, we showed that CD69 expression is upregulated also in scRNA-seq obtained from ICI-treated patients, thus providing a strong translational rational. Given the relatively short survival times of patients with GBM and the increasing number of immunotherapies being evaluated, early measurement of therapeutic efficacy can have drastic implications for clinical care, preventing unnecessary side effects of ineffective therapies, allowing patients more time to receive alternative therapeutic regimens, and providing validity for continuing treatment in responding patients. We envision clinically, patients may serve as their own baseline, and pretreatment PET scan may serve as appropriate baseline to assess the increase in PET signal upon ICI. Overall, given our promising results, we believe that CD69 immuno-PET imaging should be further developed for clinical evaluation and tested in other immunotherapies to assess its ability to be used as a general assessment tool for immunotherapy response.

## Supplementary Material

Supplementary Figure 1 CD69 is upregulated upon T-cell activation.

Supplementary Figure 2 CD69 validation by tissue staining.

Supplementary Figure 3 CD69 analysis in single-cell RNA-seq data.

Supplementary Figure 4 Production, quality control, and immunoreactivity of 89Zr-DFO-CD69 Ab.

Supplementary Figure 5 Equilibrium dissociation constants of anti-CD69 Abs clone CD69.2.2 and H1.2F3.

Supplementary Figure 6 SUV analysis and BioD data of CD69 immuno-PET.

Supplementary Figure 7 Validation of CD69 expression on immune cells by flow cytometry.

Supplementary Figure 8 MRI analysis of tumor volume prior to immuno-PET.

Supplementary Figure 9 Pearson's correlation coefficients analysis between immuno-PET signals and survival in ICI and control groups.

Supplementary Figure 10 MRI analysis of tumor volume before and after treatment.
