# Supplementary figures and images for: Immuno-PET Imaging of CD69 Visualizes T-Cell Activation and Predicts Survival Following Immunotherapy in Murine Glioblastoma
Source: Cancer Res Commun. 2023 Jul 6;3(7):1173–88. doi: 10.1158/2767-9764.CRC-22-0434 (PMC10324623; doi:10.1158/2767-9764.CRC-22-0434)

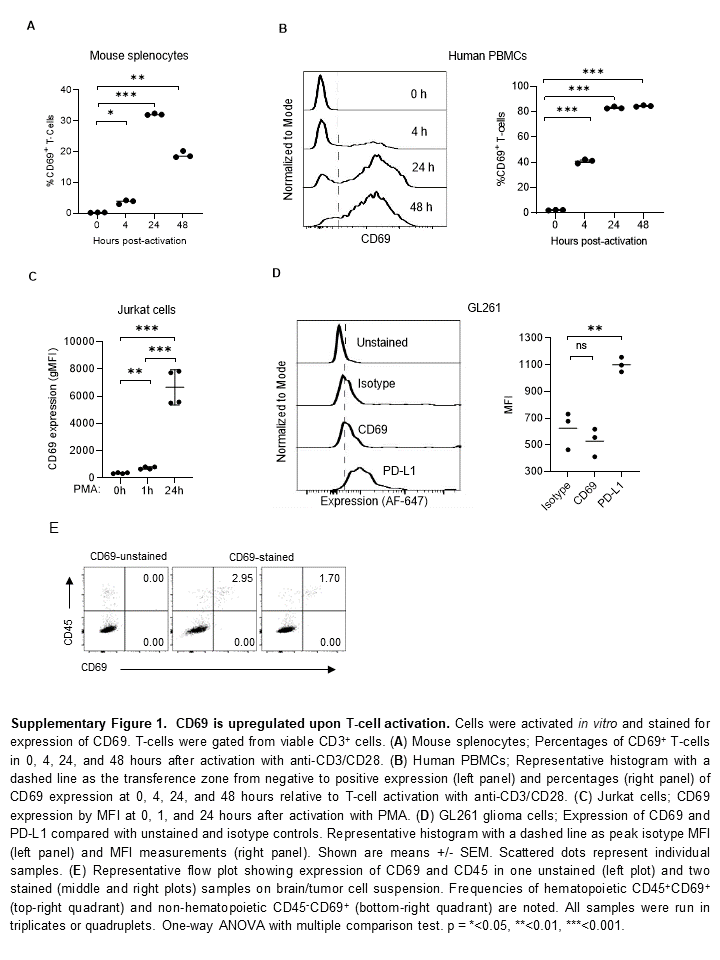

Supplement: Supplementary Figure 1 — CD69 is upregulated upon T-cell activation. [file crc-22-0434-s01.png]

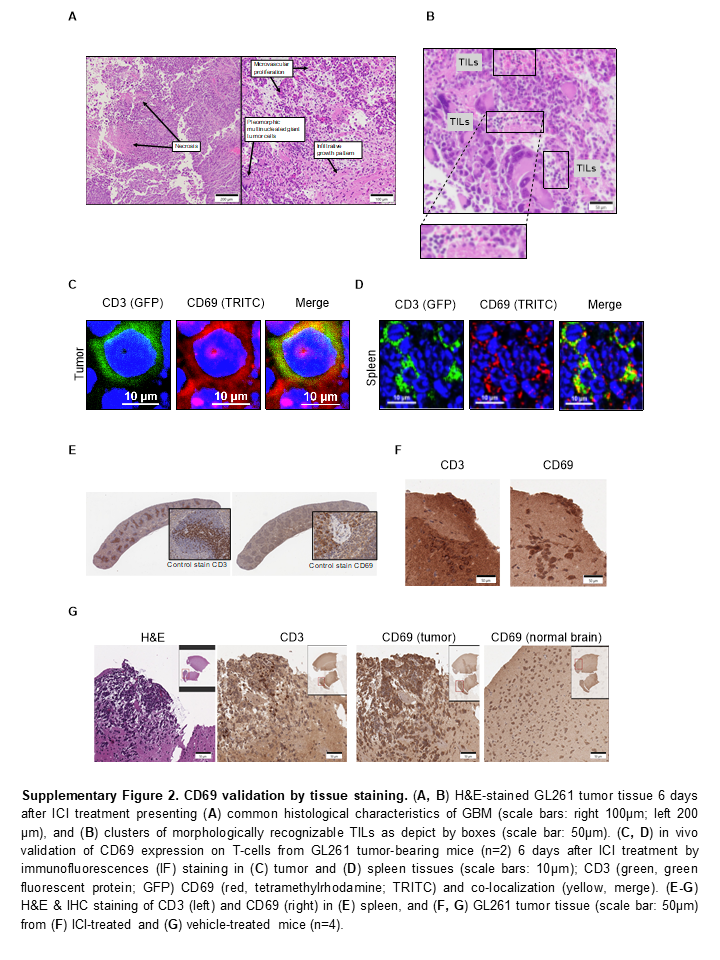

Supplement: Supplementary Figure 2 — CD69 validation by tissue staining. [file crc-22-0434-s02.png]

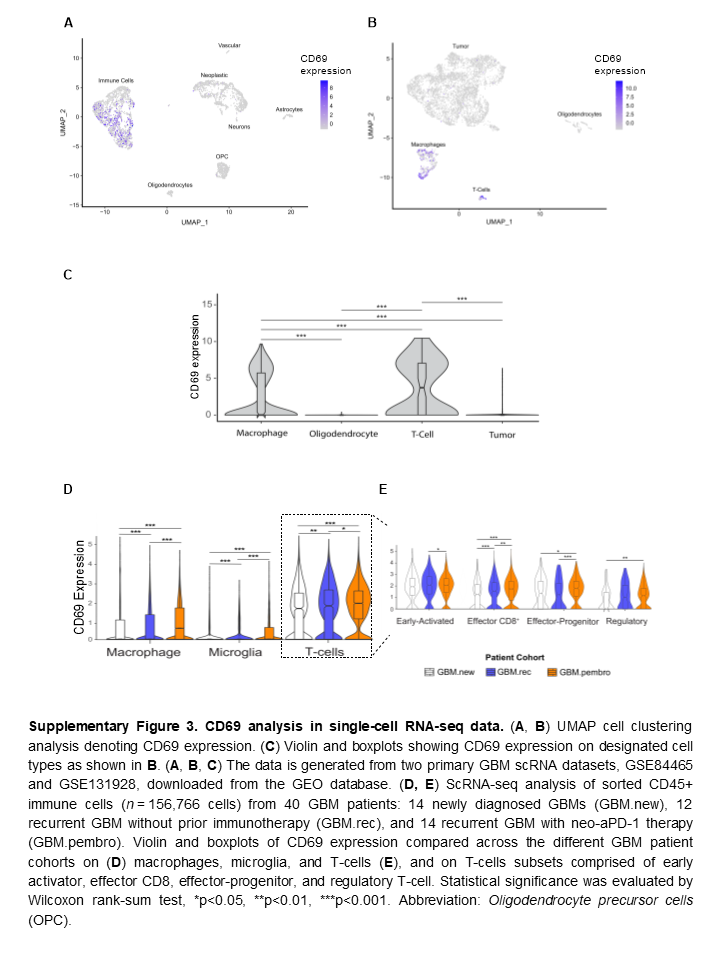

Supplement: Supplementary Figure 3 — CD69 analysis in single-cell RNA-seq data. [file crc-22-0434-s03.png]

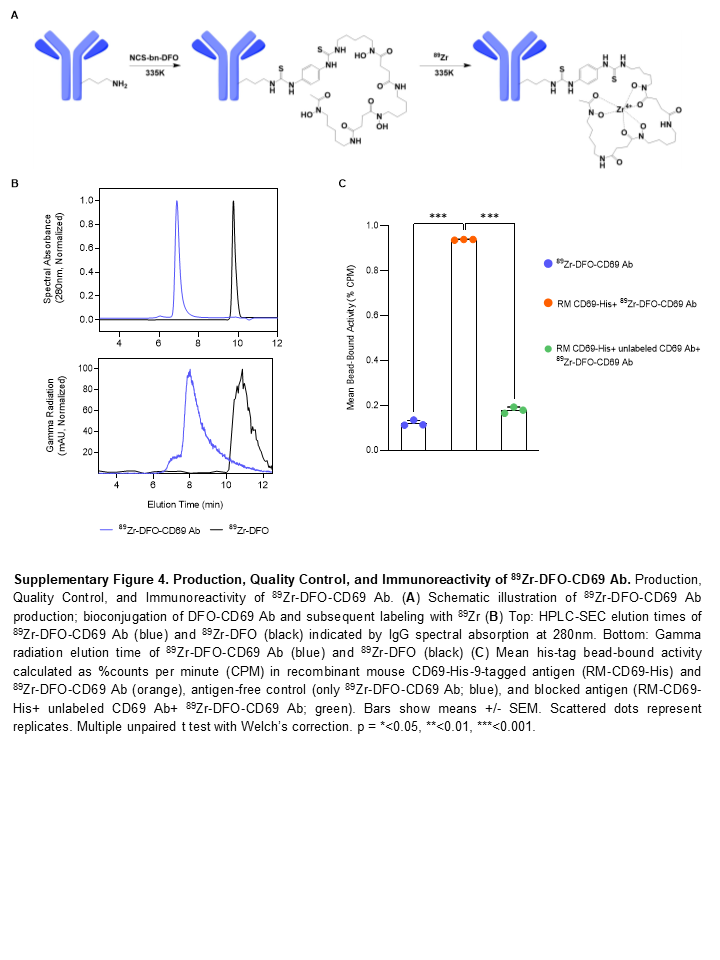

Supplement: Supplementary Figure 4 — Production, quality control, and immunoreactivity of 89Zr-DFO-CD69 Ab. [file crc-22-0434-s04.png]

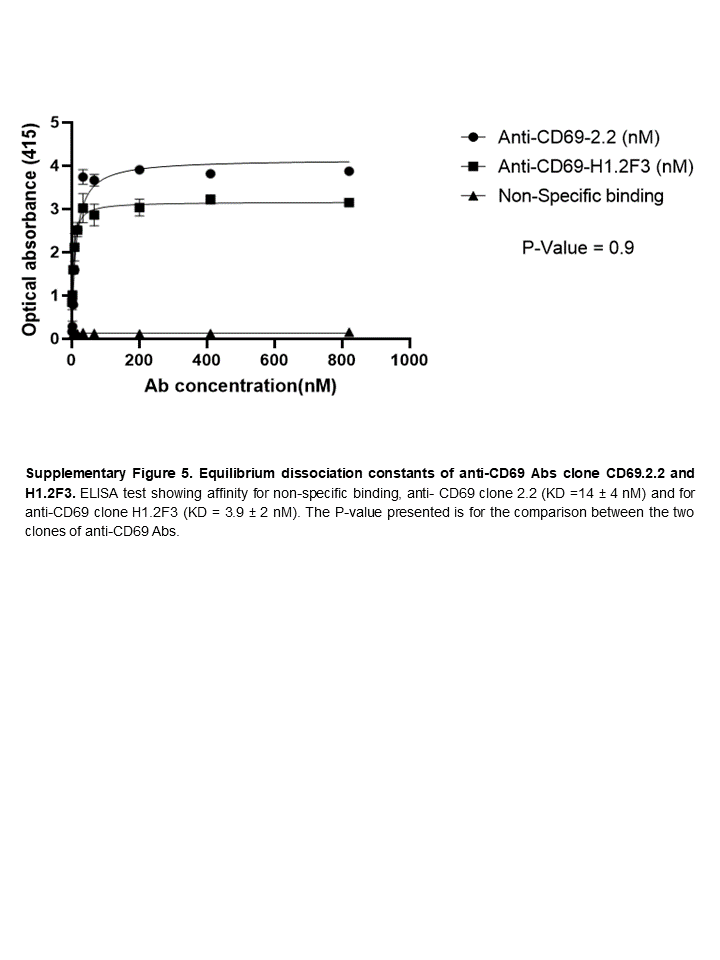

Supplement: Supplementary Figure 5 — Equilibrium dissociation constants of anti-CD69 Abs clone CD69.2.2 and H1.2F3. [file crc-22-0434-s05.png]

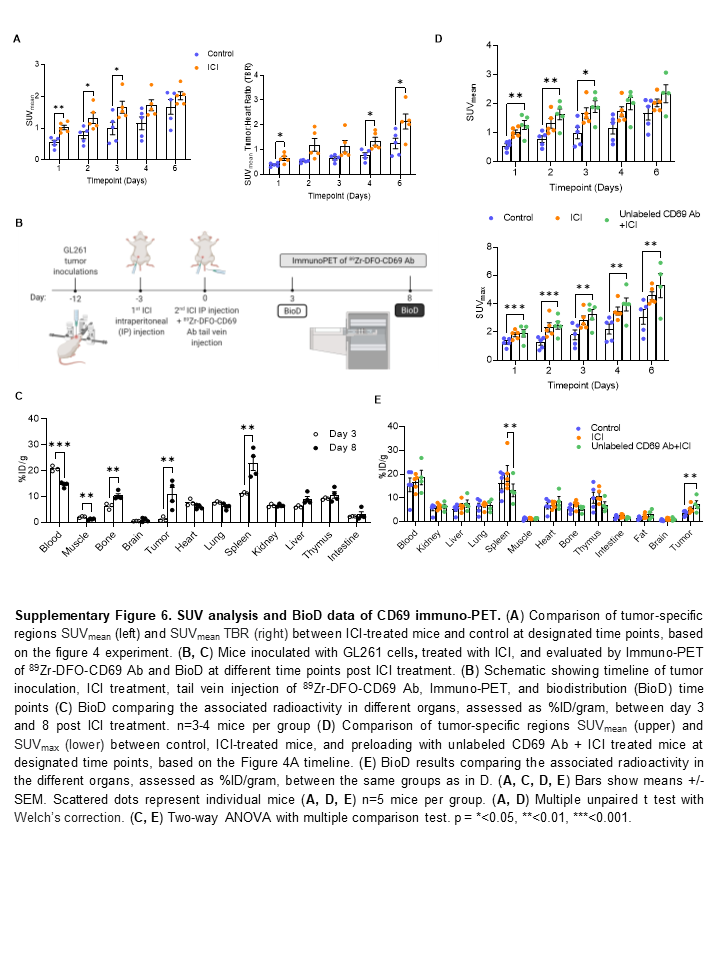

Supplement: Supplementary Figure 6 — SUV analysis and BioD data of CD69 immuno-PET. [file crc-22-0434-s06.png]

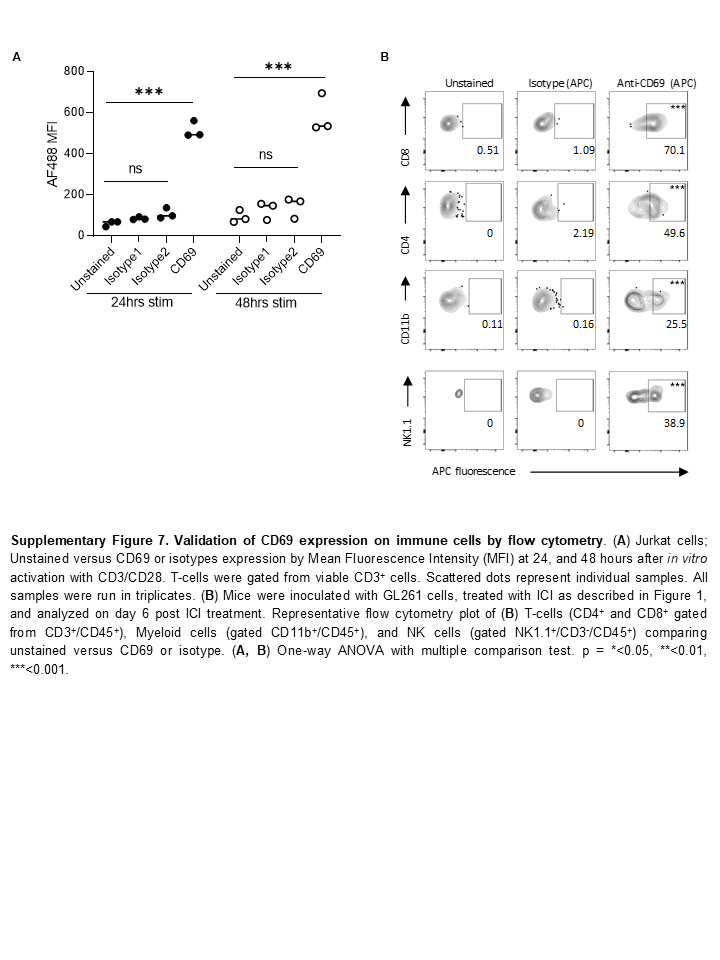

Supplement: Supplementary Figure 7 — Validation of CD69 expression on immune cells by flow cytometry. [file crc-22-0434-s07.png]

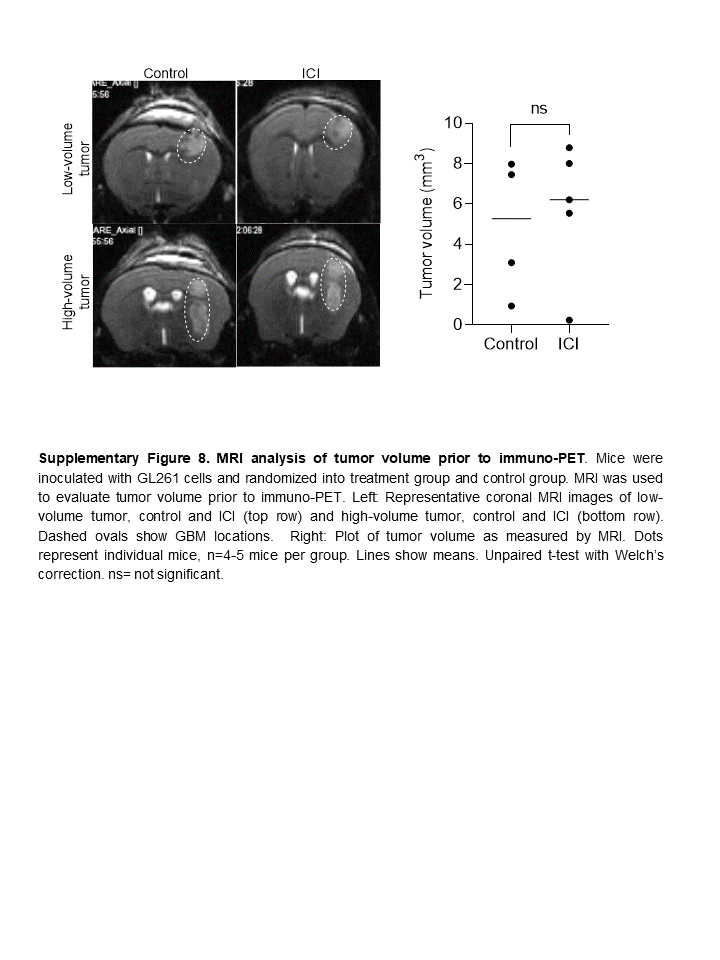

Supplement: Supplementary Figure 8 — MRI analysis of tumor volume prior to immuno-PET. [file crc-22-0434-s08.png]

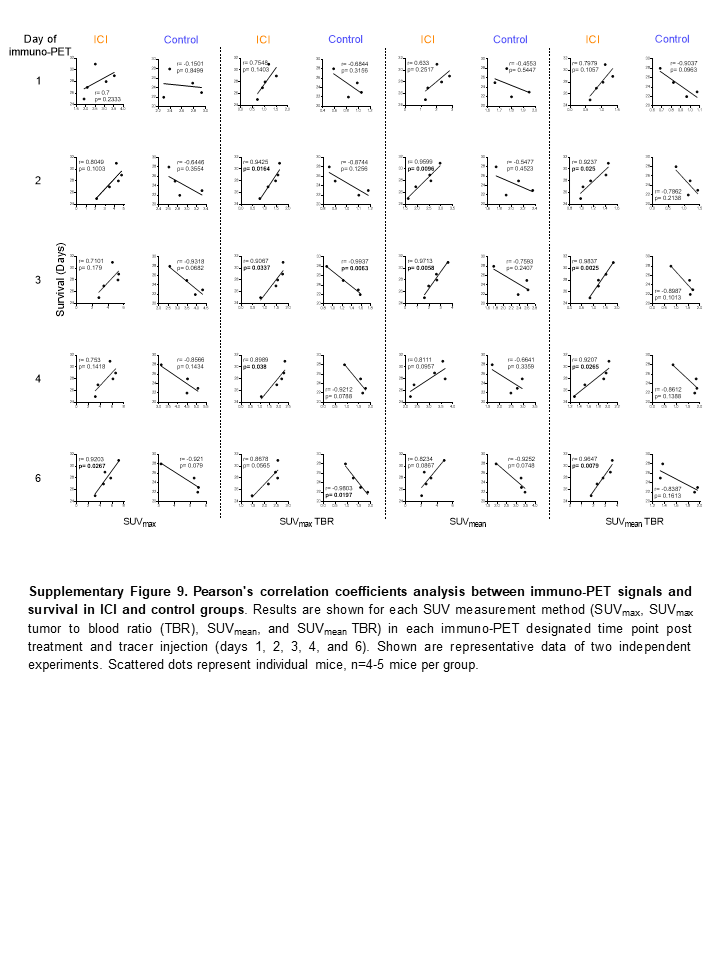

Supplement: Supplementary Figure 9 — Pearson's correlation coefficients analysis between immuno-PET signals and survival in ICI and control groups. [file crc-22-0434-s09.png]

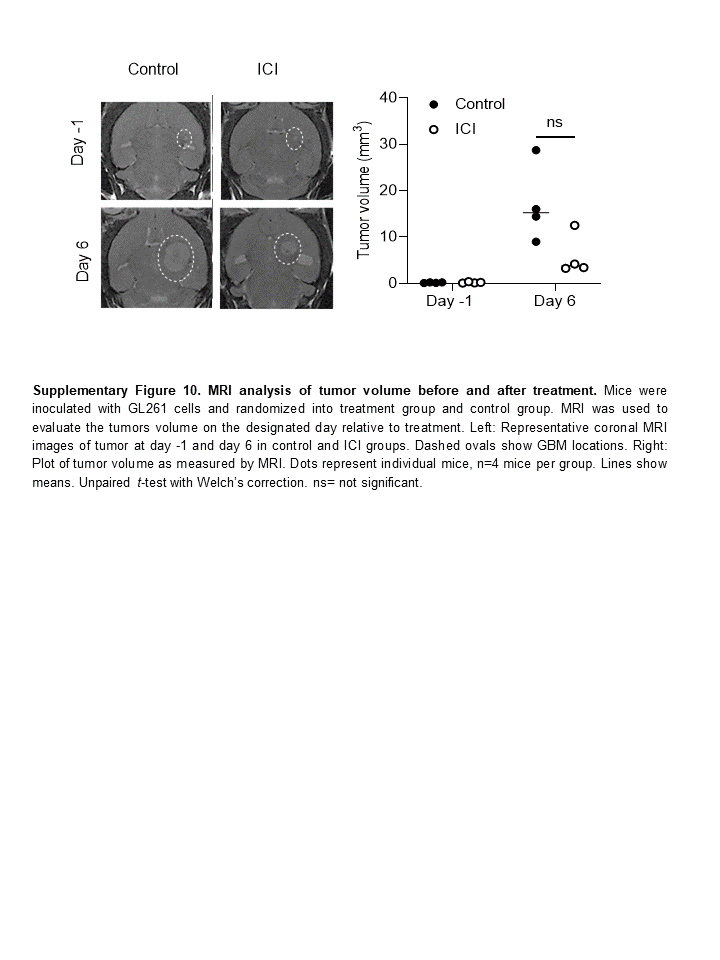

Supplement: Supplementary Figure 10 — MRI analysis of tumor volume before and after treatment. [file crc-22-0434-s10.png]
